# Exploring hardware implementation feasibility of post-quantum cryptography in embedded systems: Evaluation of NIST-standardized ML-KEM, ML-DSA and SLH-DSA on ESP32-C6

**DOI:** 10.1371/journal.pone.0355179

**Published:** 2026-08-18

**Authors:** Daniel Patryk Karcz, Marcin Niemiec, Michail Alexandros Kourtis, Tobias Köppl

**Affiliations:** 1 AGH University of Krakow, Faculty of Computer Science, Electronics and Telecommunications, Krakow, Poland; 2 National Center for Scientific Research "Demokritos", Athens, Greece; 3 Fraunhofer Institute for Open Communication Systems (FOKUS), Berlin, Germany; China Medical University Taiwan, TAIWAN

## Abstract

The transition to post-quantum cryptography (PQC) raises practical concerns regarding the feasibility of standardized algorithms on resource-constrained embedded platforms. While National Institute of Standards and Technology (NIST)-selected PQC schemes are designed for broad applicability, their real-world performance on low-power microcontrollers remains insufficiently characterized. Using a configurable on-device evaluation framework, we assess the lattice-based key encapsulation mechanism (ML-KEM), lattice-based digital signature scheme (ML-DSA), and the hash-based digital signature scheme (SLH-DSA) implementations from the liboqs library across all NIST-standardized parameter sets and security levels. Our measurements focus on execution latency, key and signature material sizes, and dynamic memory behavior under realistic embedded constraints. Experimental results show that all evaluated ML-KEM, ML-DSA, and SLH-DSA parameter sets execute successfully on the ESP32-C6 without external memory support. ML-KEM exhibits stable and predictable memory usage across parameter sets, with execution time increasing proportionally with the security level. ML-DSA key generation and signing operations incur higher computational cost, while verification remains comparatively efficient. Evaluated SLH-DSA parameter sets demonstrate substantially higher execution latency, reflecting the inherent computational cost of hash-based signature schemes on embedded hardware. Overall, our results indicate that selected NIST PQC algorithms are deployable on modern embedded microcontrollers, provided that performance and latency trade-offs are carefully considered. The presented evaluation framework and empirical results provide practical guidance for the design of post-quantum secure embedded systems.

## Introduction

The emergence of quantum computing poses a significant threat to classical public-key cryptographic schemes such as RSA (Rivest–Shamir–Adleman) and elliptic-curve cryptography (ECC), as Shor’s algorithm can break their underlying mathematical assumptions in polynomial time [1]. Grover’s algorithm further reduces the effective security of symmetric primitives, motivating larger key sizes such as the Advanced Encryption Standard (AES) with 256-bit keys in the post-quantum era [2]. As long-lived and widely deployed systems—including Internet-of-Things infrastructures, industrial controllers and secure boot mechanisms—depend on cryptographic trust anchors, preparing for migration towards quantum-resistant cryptography is becoming an urgent requirement.

Post-quantum cryptography addresses this problem by introducing schemes that remain secure against adversaries equipped with quantum computers. The National Institute of Standards and Technology of the United States of America has standardized three primary post-quantum cryptographic algorithms in Federal Information Processing Standards (FIPS) 203–205: ML-KEM for key encapsulation [3], ML-DSA for digital signatures [4], and SLH-DSA as a stateless hash-based signature scheme [5]. While these algorithms demonstrate excellent performance on general-purpose computing platforms, their execution cost on low-power embedded devices remains insufficiently explored. Many microcontrollers operate with limited RAM, constrained compute capability, and without hardware acceleration beyond basic AES or Secure Hash Algorithm (SHA) primitives [6], raising concerns about the feasibility of post-quantum cryptography in real-world embedded deployments.

Existing research primarily evaluates lattice-based key encapsulation mechanisms or digital signature schemes in isolation, often on platforms equipped with external pseudo-static RAM (PSRAM) or more powerful processors. Hash-based signature schemes such as SLH-DSA are frequently omitted due to their substantial computational cost [7,8]. This motivates a systematic evaluation of all NIST-standardized post-quantum cryptographic algorithms on genuinely constrained microcontrollers.

In this work, we explore the feasibility of executing NIST-standardized post-quantum cryptographic schemes on the ESP32-C6 microcontroller. We port the liboqs library to the ESP-IDF environment [9] by maintaining a dedicated liboqs branch that is developed as an integral part of our embedded evaluation framework. This approach enables systematic development, future optimization, execution, and measurement of post-quantum cryptographic algorithms on constrained hardware. Using benchmarking modules that are part of our evaluation framework, we measure execution time and dynamic memory usage and evaluate the complete set of NIST-standardized parameter sets for ML-KEM, ML-DSA, and SLH-DSA under realistic hardware constraints. Our results quantify the practical execution cost of post-quantum cryptography on devices without external memory and identify which algorithms are suitable for on-device use today, which remain viable primarily for offline or infrequent operations, and which require further implementation-level optimizations to enable broader practical deployment on embedded platforms.

Our contributions, carried out within the framework of a European research project [10], are as follows:

- Design and implementation of an embedded evaluation framework for post-quantum cryptography on constrained microcontrollers, including a dedicated liboqs branch developed as an integral part of the framework.
- Full on-device execution and evaluation of all NIST-standardized ML-KEM, ML-DSA, and SLH-DSA parameter sets on the ESP32-C6 microcontroller without external RAM.
- Detailed measurement of execution time and dynamic memory usage for key generation, encapsulation or signing, and decapsulation or verification under both baseline and realistic embedded workload conditions.
- A comparative analysis contrasting the ESP32-C6 results with representative embedded PQC implementations reported for ARM Cortex-M4, RISC-V, and TriCore-based platforms, providing architectural and implementation-level context for embedded deployment.
- Evaluation of representative ML-KEM and ML-DSA combinations to approximate the computational and memory requirements of complete post-quantum security protocols.
- A feasibility analysis identifying practical deployment options, limitations, and optimization opportunities for post-quantum cryptography in embedded systems.
- Public release of source code and measurement logs to support reproducibility and further research.

## Background and related work

Post-quantum cryptography introduces cryptographic schemes designed to remain secure against both classical and quantum adversaries [1]. Quantum attacks relevant to modern cryptography fall into two main categories. Shor’s algorithm enables polynomial-time attacks against widely deployed public-key schemes such as RSA and elliptic-curve cryptography, while Grover’s algorithm provides a quadratic speedup for brute-force search against symmetric primitives and hash functions, effectively reducing their security margin by half [2].

## NIST standardization

To address the threat posed by quantum-capable adversaries to public-key infrastructures, NIST standardized three primary post-quantum cryptographic algorithms in FIPS 203–205 [11]: ML-KEM for key encapsulation, ML-DSA for digital signatures, and SLH-DSA as a stateless hash-based signature scheme [3–5]. These standards represent the first generation of production-ready post-quantum cryptographic primitives intended for broad deployment across diverse application domains.

For clarity, Table 1 summarizes the mapping between NIST-standardized post-quantum cryptographic algorithm names used throughout this paper and their corresponding pre-standard names commonly found in the literature.

**Table 1:** Mapping between NIST-standardized and pre-standard post-quantum cryptographic algorithms.

NIST name	Pre-standard name	Primitive	FIPS
ML-KEM	Kyber	Key encapsulation mechanism	FIPS 203
ML-DSA	Dilithium	Digital signature algorithm	FIPS 204
SLH-DSA	SPHINCS+	Digital signature algorithm	FIPS 205

## Characteristics of ML-KEM, ML-DSA and SLH-DSA

ML-KEM and ML-DSA are lattice-based constructions relying on the hardness of the Module Learning With Errors (Module-LWE) and Module Short Integer Solution (Module-SIS) problems, respectively. These schemes offer relatively compact key material and signatures while demonstrating strong performance on general-purpose computing platforms. As a result, they are widely considered natural candidates for replacing classical public-key primitives in secure communication and authentication protocols.

SLH-DSA is a stateless hash-based digital signature scheme constructed from Merkle trees and Winternitz one-time signatures. Its security relies solely on the properties of cryptographic hash functions, providing conservative and well-understood security assumptions [12,13]. However, this design results in significantly larger signatures and substantially higher computational effort, particularly during key generation and signing operations.

## Embedded constraints and feasibility challenges

Embedded systems introduce constraints that significantly complicate the deployment of post-quantum cryptographic primitives. Typical microcontrollers operate with limited on-chip memory, moderate CPU frequency, and without specialized hardware acceleration for lattice-based arithmetic or for the hash-intensive operations required by stateless hash-based signature schemes. In addition, memory resources must be shared between application code, operating system services, and cryptographic workloads.

These constraints are particularly relevant for post-quantum cryptography, as many standardized algorithms were primarily designed and evaluated with general-purpose computing platforms in mind [14]. When executed on constrained microcontrollers, both execution time and dynamic memory consumption may therefore become limiting factors for practical deployment.

Consequently, systematic evaluation of execution time, memory footprint, and overall feasibility of post-quantum cryptographic algorithms on resource-constrained microcontrollers is essential for assessing their suitability for real-world embedded applications, as addressed in this study.

## Related work

Post-quantum cryptography has been extensively studied on general-purpose computing platforms, including desktop, workstation, and server-class environments [1,2]. In contrast, its deployment on constrained embedded platforms remains an active area of research. Existing studies primarily focus on selected algorithms, reduced parameter sets, or platforms equipped with external memory, leaving important feasibility questions unanswered for resource-limited microcontrollers.

### Lattice-based cryptography on embedded platforms.

Several works have investigated the performance of lattice-based post-quantum algorithms on embedded systems. Fritzmann et al. [15] evaluated key encapsulation mechanisms such as CRYSTALS-Kyber and Saber in the context of automotive security using Infineon AURIX microcontrollers. Their results demonstrated technical feasibility but identified execution time and memory overhead as significant challenges. Similar observations were reported in studies targeting ARM Cortex-M platforms, where optimized implementations of Kyber and Dilithium were shown to require careful memory management, platform-specific optimizations, and reduced buffering to fit within tight RAM constraints [14].

### Post-quantum cryptography in IoT systems.

In the IoT domain, Kratochvil [16] evaluated Kyber512 and Dilithium2 on ESP32-class microcontrollers, analyzing trade-offs between execution time and memory footprint. These studies highlight the importance of tailoring post-quantum implementations to highly constrained environments but typically consider only a subset of algorithms or security levels. Moreover, many reported implementations rely on external RAM, assembly-level optimizations, or omit long-running cryptographic operations that may dominate real-world behavior, particularly for hash-based signature schemes.

### Digital signature efficiency and comparative analyses.

Dziechciarz and Niemiec [17] conducted a comparative efficiency analysis of NIST-standardized digital signature schemes across multiple software and hardware environments. Their work provides valuable cross-platform insights into computational cost, key sizes, and signature sizes, but does not address end-to-end feasibility on deeply constrained microcontrollers operating without external memory or hardware acceleration.

### Hash-based signatures and SPHINCS+ optimization.

Hash-based signature schemes, particularly SPHINCS+ and its standardized variant SLH-DSA, have received limited attention in embedded evaluations due to their extreme computational cost. Bernstein et al. [18] introduced SPHINCS+ as a stateless hash-based signature scheme with conservative security assumptions, while Hülsing et al. [8] demonstrated that even signature verification can pose significant challenges under severe memory constraints. Niederhagen et al. [7] proposed a streaming-based SPHINCS+ implementation for Trusted Platform Modules (TPMs), significantly reducing memory usage at the cost of increased architectural complexity. While effective in specialized hardware, such approaches are not directly transferable to general-purpose microcontrollers without dedicated buffering or streaming support.

### Post-quantum protocols and system integration.

Beyond isolated benchmarks, several works have explored the integration of post-quantum cryptography into embedded communication protocols. Bürstinghaus-Steinbach et al. [19] demonstrated the integration of Kyber and SPHINCS+ into the mbedTLS library to enable post-quantum TLS on embedded devices. Further evaluations of post-quantum TLS on embedded platforms highlighted increased handshake latency, memory consumption, and code size when integrating lattice-based and hash-based primitives into full protocol stacks [20]. These results underscore that protocol-level feasibility depends not only on cryptographic runtime but also on system integration and resource sharing.

### Positioning of this work.

Building upon prior research, this work provides the first comprehensive end-to-end evaluation of all three NIST-standardized post-quantum cryptographic algorithms—ML-KEM, ML-DSA, and SLH-DSA—on a single constrained embedded platform. In contrast to existing studies that focus on selected schemes, reduced parameter sets, or rely on external memory, the evaluation targets an ESP32-C6 microcontroller based on a 32-bit RISC-V core, operating without PSRAM support and without hardware acceleration for post-quantum primitives. Although the platform provides hardware accelerators for standard cryptographic building blocks such as SHA and AES, no dedicated acceleration for ML-KEM, ML-DSA, or SLH-DSA is available. All standardized parameter sets are considered, including SHA2- and SHAKE-based SLH-DSA variants, enabling a systematic analysis of execution time, dynamic memory behavior, and practical feasibility limits in realistic embedded deployments.

## Materials and methods

This section describes the hardware and software components used in the experiments. First of all, the ESP32-C6 was selected as a representative platform for constrained embedded deployments due to its wide availability, low cost, frequent use in academic and experimental embedded systems research, and increasing adoption in IoT and edge-security applications.

### Hardware platform

All experiments were conducted on the ESP32-C6 microcontroller, a low-cost system-on-chip based on a single-core 32-bit RISC-V processor. The device operates at a maximum clock frequency of 160 MHz and provides 512 KB of on-chip SRAM, with no support for external PSRAM. This memory is shared between application code, stack, heap, and the real-time operating system. The ESP32-C6 includes hardware accelerators for symmetric cryptographic primitives (AES and SHA) as well as an on-chip true random number generator (TRNG); however, no hardware support for post-quantum cryptographic primitives is available. The key hardware characteristics of the ESP32-C6 platform relevant to this study are summarized in Table 2.

**Table 2:** Hardware characteristics of the ESP32-C6 microcontroller used in this study.

Component	Specification
Microcontroller	ESP32-C6
CPU architecture	32-bit RISC-V (single-core)
Maximum clock frequency	160 MHz
On-chip SRAM	512 KB
External RAM support	None
Non-volatile memory	External flash (SPI)
Hardware cryptographic accelerators	AES, SHA
True random number generator	On-chip TRNG

### Software stack

The software stack is based on Espressif’s ESP-IDF framework (version 5.5.1), with FreeRTOS used as the integrated real-time operating system. Post-quantum cryptographic algorithms were provided by the Open Quantum Safe liboqs library (version 0.14.0).

The firmware was compiled using the riscv32-esp-elf-gcc toolchain (GCC 14.2.0, crosstool-NG esp-14.2.0_20241119) distributed with ESP-IDF. The build configuration used the ESP-IDF default debug optimization profile, corresponding to

CONFIG_COMPILER_OPTIMIZATION_DEBUG=y
CONFIG_COMPILER_OPTIMIZATION_LEVEL_DEBUG=y

The same ESP-IDF version, compiler toolchain, liboqs version, and build configuration were used for all reported experiments to ensure consistency and reproducibility.

**Fig 1:**
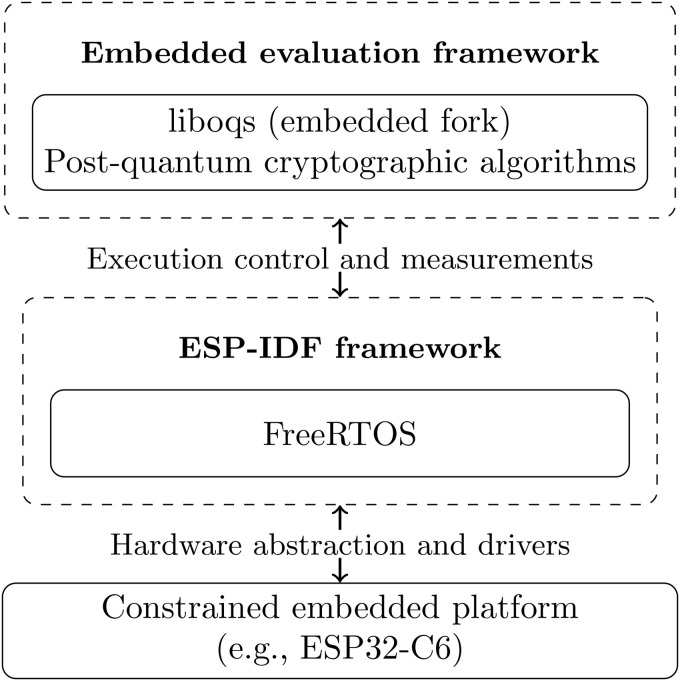
Structure of the embedded evaluation framework and its integration within the ESP-IDF ecosystem. The evaluation framework encapsulates the embedded liboqs integration and benchmarking logic, while ESP-IDF provides the integrated FreeRTOS runtime and hardware abstraction for execution on constrained embedded platforms.

Since the upstream liboqs library is not directly compatible with resource-constrained embedded environments, the evaluation presented in this work is conducted using a dedicated embedded evaluation framework that integrates liboqs into the ESP-IDF ecosystem. The framework provides a structured environment for on-device execution, configuration, measurement, and future optimization of post-quantum cryptographic algorithms on constrained embedded platforms. The structure of the embedded evaluation framework and its integration within the ESP-IDF ecosystem are illustrated in Fig 1.

To reduce code size and memory footprint, liboqs was configured in a minimal build mode, enabling only the evaluated algorithms and parameter sets. All unused cryptographic schemes, test utilities, and architecture-specific optimizations not applicable to the RISC-V architecture were disabled. No OpenSSL dependencies were included.

Randomness required by the evaluated algorithms was obtained directly from the ESP32-C6 on-chip TRNG via a custom liboqs RNG callback implemented using the ESP-IDF esp_random() interface, which sources entropy exclusively from the hardware random number generator. As a result, no software-based pseudo-random number generator or deterministic random bit generator was used during cryptographic key generation or signing operations. The evaluation framework therefore obtains randomness directly from the hardware entropy source rather than from a software DRBG seeded by the TRNG. Although random number generation is required during key generation and certain signing operations, its contribution to the overall execution time is expected to be negligible compared to the computational cost of the evaluated PQC algorithms, which is dominated by polynomial arithmetic, hashing, and signature processing. Consequently, the use of the hardware TRNG is not expected to significantly influence the reported benchmark results.

### Benchmark harness

As part of the proposed post-quantum cryptographic evaluation framework, a dedicated benchmark harness was implemented to measure the performance and memory behavior of individual cryptographic operations in isolation.

For each algorithm and parameter set, the harness performs the full sequence of cryptographic operations using the official liboqs API. For key encapsulation mechanisms, this includes key generation, encapsulation, and decapsulation. For digital signature schemes, key generation, signing, and verification are executed.

The benchmark harness was designed to minimize measurement interference. Timing measurements were performed exclusively around cryptographic function calls, excluding all I/O and logging operations. Results were emitted both in a machine-readable CSV format and as human-readable console output.

The benchmark harness constitutes only one component of the broader evaluation framework, which additionally encompasses configuration management, algorithm selection, and result post-processing.

### Measurement methodology

Baseline benchmarks were executed within a single FreeRTOS task context to minimize interference from unrelated system activity. The CPU frequency was fixed at 160 MHz. For the baseline measurements, wireless peripherals were disabled to isolate the computational characteristics of the evaluated cryptographic algorithms. Additional experiments were performed under realistic deployment conditions, including concurrent application workloads and active wireless communication subsystems (Wi-Fi and Bluetooth Low Energy), to evaluate their impact on cryptographic performance.

Execution time was measured using the ESP-IDF high-resolution timer with microsecond precision. For each cryptographic operation *O*, runtime was computed as


$$\begin{array}{c} t_{O}=t_{\text{end}}-t_{\text{start}}, \end{array}$$

where timestamps were recorded immediately before and after the corresponding liboqs cryptographic function call. For ML-KEM, ML-DSA, and combined ML-KEM/ML-DSA scenarios, each cryptographic operation was measured over 100 independent runs. Due to the substantially longer execution times of SLH-DSA operations, the number of measurement repetitions was reduced and selected according to the evaluated scenario. Reported results correspond to the mean execution time, with variability expressed using the standard deviation. Dynamic memory usage was monitored using the ESP-IDF heap introspection API. Available heap memory was sampled before and after each cryptographic operation to estimate peak dynamic memory consumption and heap fragmentation behavior during execution. In addition to runtime and dynamic memory measurements, program memory footprint (flash memory usage) was analyzed for different algorithm configurations to assess deployment feasibility on resource-constrained embedded devices. Moreover, all message sizes were intentionally kept small and constant to isolate algorithmic overhead from message-dependent effects.

### Evaluated algorithms

The evaluation covers all NIST-standardized post-quantum cryptographic algorithms specified in FIPS 203–205. For ML-KEM and ML-DSA, all standardized parameter sets were evaluated, corresponding to their respective NIST security levels. For SLH-DSA, both SHA2- and SHAKE-based variants were tested, including configurations optimized for smaller signatures (“s”) and faster signing (“f”). In addition to standalone algorithm benchmarks, combined ML-KEM and ML-DSA deployment scenarios were evaluated to better reflect practical embedded system operation, where key establishment and digital signature mechanisms are often used together within the same application workflow.

All algorithms were evaluated on the ESP32-C6 platform without external memory. Furthermore, additional experiments were conducted to assess the impact of the ESP32-C6 hardware SHA accelerator on the performance of selected cryptographic operations, particularly for hash-intensive algorithms such as SLH-DSA.

It is worth mentioning that ML-DSA parameter sets correspond to NIST security levels 2, 3, and 5, whereas ML-KEM and SLH-DSA follow security levels 1, 3, and 5. Due to the large number of standardized SLH-DSA variants, only representative parameter sets for each security level are shown in Table 3. The complete list of evaluated SHA2- and SHAKE-based SLH-DSA variants is provided in S1 Table.

**Table 3:** Post-quantum cryptographic algorithms evaluated in this study. For each algorithm family, representative standardized parameter sets and corresponding NIST security levels are reported.

Algorithm family	Parameter set	Primitive	NIST level
ML-KEM	ML-KEM-512	Key encapsulation mechanism	1
ML-KEM	ML-KEM-768	Key encapsulation mechanism	3
ML-KEM	ML-KEM-1024	Key encapsulation mechanism	5
ML-DSA	ML-DSA-44	Digital signature	2
ML-DSA	ML-DSA-65	Digital signature	3
ML-DSA	ML-DSA-87	Digital signature	5
SLH-DSA	SLH-DSA-128	Digital signature	1
SLH-DSA	SLH-DSA-192	Digital signature	3
SLH-DSA	SLH-DSA-256	Digital signature	5

### Benchmark input data

For signature benchmarks, a fixed-length message was used as input. For ML-DSA, the message was “Test message for ML-DSA benchmark” with a length of 33 bytes. For SLH-DSA, the message was “Hello PQ world!” with a length of 15 bytes. All messages consisted of ASCII characters and were constant across all runs for a given algorithm family.

ML-KEM does not process application messages, as encapsulation produces a ciphertext and a fixed-length shared secret. Therefore, message size is not applicable for ML-KEM benchmarks.

## Results

This section presents experimental results obtained on the ESP32-C6 microcontroller for all evaluated post-quantum cryptographic algorithms. Execution time and dynamic memory behavior are reported for each cryptographic operation, including key generation, encapsulation or signing, and decapsulation or verification, depending on the algorithm class.

To ensure clarity and comparability, results are grouped by algorithm family. For each family, performance trends across standardized parameter sets are analyzed with respect to increasing NIST security levels. In addition to the minimum available heap memory, the largest free heap block was monitored throughout the benchmark campaigns as an indicator of heap fragmentation. No observable fragmentation effects were detected for any of the evaluated algorithms. This behavior is likely attributable to the memory management characteristics of the evaluated liboqs implementations, which primarily allocate temporary buffers during cryptographic operations and release them upon completion, thereby limiting long-term heap fragmentation. In addition to raw execution time, minimum available heap memory observed during execution is reported to assess practical feasibility under strict memory constraints. Unless stated otherwise, all measurements were conducted under identical experimental conditions as described in the Materials and Methods section.

Two realistic deployment scenarios were evaluated. The first scenario consisted of periodic on-chip temperature acquisition, background sensor-data processing, and Bluetooth Low Energy (BLE) telemetry. The sensor task sampled the internal temperature sensor at 1 Hz, while the processing stage computed a moving average, minimum and maximum values, and a trend indicator, introducing a controlled lightweight CPU load. The processed state was periodically transmitted through a custom BLE GATT characteristic during cryptographic execution.

The second scenario extended the same workload by enabling Wi-Fi connectivity and maintaining an active connection to a wireless network throughout the measurements. This configuration allowed evaluation of the additional impact of wireless subsystem activity, background driver tasks, interrupt handling, and resource contention on cryptographic performance. Both scenarios were designed to represent realistic lightweight IoT deployment conditions rather than artificial stress-test environments.

### ML-KEM performance

This subsection summarizes the performance characteristics of ML-KEM on the ESP32-C6 platform, including standardized key material sizes, execution time, and dynamic memory usage across all three NIST-defined parameter sets.

As expected, increasing the security level from ML-KEM-512 to ML-KEM-768 and ML-KEM-1024 results in larger key and ciphertext sizes, reflecting the higher computational and memory requirements associated with stronger security parameters (Table 4).

**Table 4:** ML-KEM object sizes for standardized parameter sets (bytes). Public key, secret key, ciphertext, and shared secret sizes as defined by the NIST-standardized ML-KEM parameter sets and obtained via the liboqs API metadata.

Parameter set	Public key	Secret key	Ciphertext	Shared secret
ML-KEM-512	800	1632	768	32
ML-KEM-768	1184	2400	1088	32
ML-KEM-1024	1568	3168	1568	32

Table 5 summarizes ML-KEM performance under three execution scenarios: an isolated baseline configuration, a realistic embedded workload with concurrent sensor processing and BLE communication, and an extended scenario that additionally maintains an active Wi-Fi connection.

**Table 5:** ML-KEM execution time and dynamic memory usage on ESP32-C6 under baseline and workload scenarios. Mean execution time (ms, mean ± SD over 100 runs) and minimum observed heap availability (bytes) during each benchmark case.

Algorithm	KeyGen (ms)	Encap (ms)	Decap (ms)	Min free heap (B)
**Baseline**				
ML-KEM-512	103.153±2.542	106.640±0.003	129.405±0.002	331.348
ML-KEM-768	170.962±2.086	170.789±0.002	204.910±0.003	329.876
ML-KEM-1024	262.557±5.854	264.323±0.003	309.833±0.002	328.244
**Workload + BLE**				
ML-KEM-512	108.904±11.050	113.138±11.424	137.115±12.327	250.808
ML-KEM-768	182.237±14.533	181.058±13.761	216.920±14.943	249.336
ML-KEM-1024	278.155±17.317	296.459±16.651	343.881±17.247	247.704
**Workload + BLE + Wi-Fi**				
ML-KEM-512	110.973±12.250	114.417±11.596	138.346±12.404	125.088
ML-KEM-768	183.672±14.754	183.099±14.119	219.474±15.197	123.616
ML-KEM-1024	280.822±16.500	283.431±16.621	331.960±17.311	121.896

Across all scenarios, execution time increases consistently with the selected security level. ML-KEM-512 exhibits the lowest computational cost, whereas ML-KEM-1024 requires approximately 2.5 times longer execution time due to the increased parameter dimensions associated with higher security levels.

The introduction of concurrent application workloads and wireless communication results in a measurable performance overhead. Compared with the baseline configuration, the workload + BLE scenario increases execution times by approximately 5–15%, depending on the operation and parameter set. Enabling Wi-Fi in addition to BLE introduces a further but relatively small increase in execution time, indicating that the cryptographic workload remains primarily CPU-bound even in the presence of active wireless subsystems.

Memory consumption exhibits a more pronounced effect. While the baseline measurements retain more than 328 kB of free heap memory, the workload + BLE scenario reduces the available heap to approximately 248–251 kB due to the memory requirements of the application tasks and BLE stack. When Wi-Fi is additionally enabled, the minimum free heap decreases further to approximately 122–125 kB.

Overall, the results indicate that ML-KEM maintains predictable scaling characteristics and remains practically deployable on resource-constrained embedded platforms, even when executed concurrently with typical IoT application workloads and active wireless communication subsystems.

For completeness, the largest free heap block was also monitored during all benchmark scenarios as an indicator of memory fragmentation and contiguous memory availability. Under the baseline configuration, the minimum largest free heap block remained stable at 303,104 B. When the realistic workload with BLE telemetry was enabled, this value decreased to 229,376 B, while the addition of Wi-Fi further reduced it to 104,448 B. Despite this substantial reduction in contiguous free memory, all ML-KEM parameter sets execute successfully. This indicates that the ESP32-C6 retains sufficient memory resources for practical deployment even under realistic operating conditions.

### ML-DSA performance

This subsection summarizes the performance characteristics of ML-DSA on the ESP32-C6 platform, including standardized key and signature sizes, execution time, and dynamic memory usage across all three NIST-defined parameter sets.

As expected, increasing the security level from ML-DSA-44 to ML-DSA-65 and ML-DSA-87 results in larger public and secret keys as well as significantly larger signature sizes, reflecting the higher computational and memory requirements associated with stronger security parameters (Table 6).

**Table 6:** ML-DSA key and signature sizes for standardized parameter sets (bytes). Public key, secret key, and signature sizes as defined by the NIST-standardized ML-DSA parameter sets and obtained via the liboqs API metadata.

Parameter set	Public key	Secret key	Signature
ML-DSA-44	1312	2560	2420
ML-DSA-65	1952	4032	3309
ML-DSA-87	2592	4896	4627

Table 7 shows that ML-DSA-44 exhibits the lowest computational cost across all evaluated scenarios, while ML-DSA-65 and ML-DSA-87 require substantially longer execution times due to their higher security parameters. The most computationally demanding operation is signing, whereas verification remains comparatively less expensive across all parameter sets.

**Table 7:** ML-DSA execution time and dynamic memory usage on ESP32-C6 under baseline and workload scenarios. Mean execution time (ms, mean ± SD over 100 runs) and minimum observed heap availability (bytes) during each benchmark case.

Algorithm	KeyGen (ms)	Sign (ms)	Verify (ms)	Min free heap (B)
**Baseline**				
ML-DSA-44	395.362±5.054	774.266±433.830	379.340±0.002	328.332
ML-DSA-65	716.231±0.004	1203.685±601.521	664.805±0.002	325.328
ML-DSA-87	1232.915±7.149	1735.696±498.016	1184.768±0.004	322.508
**Workload + BLE**				
ML-DSA-44	418.853±19.170	916.075±474.205	401.975±17.852	247.800
ML-DSA-65	758.688±16.297	1277.744±495.635	704.589±16.947	244.796
ML-DSA-87	1306.037±17.449	1860.525±622.458	1255.261±15.150	241.976
**Workload + BLE + Wi-Fi**				
ML-DSA-44	424.142±19.790	893.759±541.178	407.182±18.309	122.112
ML-DSA-65	768.171±16.285	1259.740±481.598	713.344±16.873	119.128
ML-DSA-87	1322.450±18.403	1958.673±756.343	1271.764±15.891	116.308

The introduction of concurrent application workloads and wireless communication results in a measurable but moderate performance degradation. Compared with the baseline configuration, the workload + BLE scenario increases execution times by approximately 5–10% for key generation and verification operations. The additional activation of Wi-Fi introduces a further but relatively small overhead, indicating that ML-DSA performance remains primarily limited by the computational complexity of the cryptographic algorithms themselves rather than by communication subsystem activity. The relatively large standard deviation observed for signing operations is attributable to the rejection-sampling procedure used during ML-DSA signature generation, which introduces inherent runtime variability between individual executions.

A substantially larger impact is observed for memory availability. The minimum free heap decreases from approximately 322–328 kB in the baseline configuration to 242–248 kB when the workload and BLE subsystem are enabled, and further decreases to approximately 116–122 kB when Wi-Fi is also active. Nevertheless, all evaluated ML-DSA parameter sets execute successfully without external memory support, demonstrating the feasibility of deploying standardized lattice-based digital signatures on the ESP32-C6 platform under realistic operating conditions.

For completeness, the largest free heap block was also monitored as an indicator of memory fragmentation and contiguous memory availability. The minimum largest free heap block decreased from 303,104 B in the baseline configuration to 225,280 B under the workload + BLE scenario and to 100,352 B when Wi-Fi was additionally enabled. Despite this reduction in contiguous free memory, no allocation failures or instability were observed during any benchmark run.

Overall, ML-DSA demonstrates predictable scaling behavior with increasing security level and remains practically deployable on resource-constrained embedded systems, particularly in scenarios where signing operations are performed infrequently while verification is executed more regularly.

### Combined ML-KEM and ML-DSA evaluation

To better reflect practical deployment scenarios, we evaluated the combined execution of key encapsulation and digital signature algorithms. Each benchmark consisted of sequential execution of ML-KEM key generation, encapsulation, and decapsulation followed by ML-DSA key generation, signing, and verification within a single measurement window. This setup approximates the behavior of embedded security protocols where key establishment and digital signatures are used together for authentication and secure communication.

The results summarized in Table 8 demonstrate that combined deployment of standardized post-quantum key encapsulation and digital signature algorithms is feasible on the ESP32-C6 platform across all evaluated security levels. Under baseline conditions, the combined execution time increased from approximately 2.0 s for the ML-KEM-512/ML-DSA-44 configuration to approximately 5.1 s for the ML-KEM-1024/ML-DSA-87 configuration, reflecting the higher computational cost associated with increasing security levels.

**Table 8:** Combined ML-KEM and ML-DSA execution on ESP32-C6. Mean execution time (ms, mean ± SD over 100 runs) and minimum observed free heap.

Configuration	Runtime (ms)	Min heap (B)
**Baseline**		
ML-KEM-512 + ML-DSA-44	1985.613±529.815	324.668
ML-KEM-768 + ML-DSA-65	3153.277±576.824	320.192
ML-KEM-1024 + ML-DSA-87	5058.269±609.632	315.740
**Workload + BLE + Wi-Fi**		
ML-KEM-512 + ML-DSA-44	2076.737±689.030	118.372
ML-KEM-768 + ML-DSA-65	3498.105±649.988	113.900
ML-KEM-1024 + ML-DSA-87	5431.395±659.706	109.520

The realistic workload scenario, including periodic temperature acquisition, background processing, BLE telemetry, and active Wi-Fi connectivity, introduced additional execution-time overhead across all configurations. Compared to the baseline measurements, total runtime increased by approximately 4.6% for ML-KEM-512/ML-DSA-44, 11.0% for ML-KEM-768/ML-DSA-65, and 7.4% for ML-KEM-1024/ML-DSA-87. The relatively large standard deviation of the combined measurements is primarily attributable to the runtime variability of the ML-DSA signing operation, which employs rejection sampling during signature generation.

The impact on memory availability was substantially more pronounced. Minimum free heap decreased from approximately 316–325 kB in the baseline configuration to approximately 110–118 kB when the complete workload and wireless communication stack were enabled. This reduction is primarily attributable to BLE and Wi-Fi subsystem memory allocation rather than to the cryptographic operations themselves.

For completeness, the minimum largest free heap block was also monitored during the combined benchmarks. Under baseline conditions, the minimum largest free heap block ranged from 294,912 B to 303,104 B, while under the full workload scenario it decreased to 90,112–102,400 B. Although the available contiguous memory region was significantly reduced by the wireless subsystems, all combined cryptographic workloads completed successfully without allocation failures or the need for external memory.

Overall, these results indicate that simultaneous deployment of ML-KEM and ML-DSA is practical on the ESP32-C6 platform, although memory availability becomes a more significant constraint than raw execution time under realistic operating conditions.

### SLH-DSA performance

This subsection presents a feasibility and performance evaluation of the hash-based SLH-DSA signature scheme on the ESP32-C6 microcontroller. Both SHA2- and SHAKE-based standardized variants were evaluated, including parameter sets optimized for smaller signatures (*s*) and faster signing (*f*).

As shown in Table 9, signature sizes increase substantially with the security category and differ significantly between the “s” (small signature) and “f” (fast signing) variants. Public and secret key sizes are independent of the selected hash function. Consequently, SHA2- and SHAKE-based SLH-DSA variants share identical key and signature sizes, with differences between profiles affecting execution time only.

**Table 9:** SLH-DSA key and signature sizes for standardized parameter sets (bytes). Public key, secret key, and signature sizes as defined by the NIST-standardized SLH-DSA parameter sets. Sizes are identical for SHA2- and SHAKE-based variants; differences between profiles affect execution time only.

Parameter set	Public key	Secret key	Signature
SLH-DSA-128s	32	64	7856
SLH-DSA-128f	32	64	17088
SLH-DSA-192s	48	96	16224
SLH-DSA-192f	48	96	35664
SLH-DSA-256s	64	128	29792
SLH-DSA-256f	64	128	49856

Tables 10 and 11 summarize the performance of SHA2- and SHAKE-based SLH-DSA variants under a realistic embedded workload consisting of periodic sensor acquisition, background processing, and BLE communication. Across all evaluated parameter sets, SLH-DSA exhibits substantially higher computational cost than the lattice-based algorithms evaluated in this study.

**Table 10:** SLH-DSA-SHA2 execution time and dynamic memory usage on ESP32-C6. Mean execution time (s, mean ± SD over 10 runs) and minimum observed free heap under a realistic embedded workload scenario consisting of sensor acquisition, background processing, and BLE communication.

Algorithm	KeyGen (s)	Sign (s)	Verify (s)	Min free heap (B)
SLH-DSA-SHA2-128s	13.645±0.031	103.778±0.030	0.107±0.012	245.748
SLH-DSA-SHA2-128f	0.212±0.016	4.954±0.015	0.298±0.020	236.516
SLH-DSA-SHA2-192s	37.856±0.017	1340.953±0.042	1.365±0.018	237.320
SLH-DSA-SHA2-192f	0.595±0.019	45.507±0.019	2.237±0.013	217.792
SLH-DSA-SHA2-256s	28.302±0.019	1619.319±0.026	1.886±0.021	223.628
SLH-DSA-SHA2-256f	1.771±0.017	99.199±0.020	2.674±0.021	203.652

**Table 11:** SLH-DSA-SHAKE execution time and dynamic memory usage on ESP32-C6. Mean execution time (s, mean ± SD over 3 runs) and minimum observed free heap under a realistic embedded workload scenario consisting of sensor acquisition, background processing, and BLE communication.

Algorithm	KeyGen (s)	Sign (s)	Verify (s)	Min free heap (B)
SLH-DSA-SHAKE-128s	1185.819±0.067	9006.762±0.036	9.023±0.324	245.972
SLH-DSA-SHAKE-128f	18.533±0.000	433.764±0.006	25.479±0.878	236.756
SLH-DSA-SHAKE-192s	1736.248±0.083	15593.018±0.252	13.062±0.176	237.576
SLH-DSA-SHAKE-192f	27.142±0.022	701.479±0.024	36.920±0.943	218.136
SLH-DSA-SHAKE-256s	1145.558±0.013	13620.301±0.052	18.749±0.102	223.964
SLH-DSA-SHAKE-256f	71.572±0.025	1438.178±0.020	38.069±0.615	203.900

For both SHA2- and SHAKE-based variants, execution time strongly depends on the selected parameter set and profile. The f” variants consistently achieve lower execution latency than the corresponding s” variants, reflecting the design trade-off between execution speed and signature size. In general, higher-security parameter sets require longer execution times, particularly for key generation and signing operations.

The difference between SHA2- and SHAKE-based variants is especially pronounced. While SHA2-based configurations require execution times ranging from fractions of a second to tens of minutes depending on the operation and parameter set, SHAKE-based variants exhibit execution times that are one to two orders of magnitude higher. Signing is by far the most computationally expensive operation across all evaluated configurations. The highest signing latency was observed for SLH-DSA-SHAKE-192s, exceeding 15,500s under the evaluated workload conditions. Notably, execution time does not increase strictly with the claimed NIST security level. For example, SLH-DSA-SHAKE-192s exhibited higher signing latency than SLH-DSA-SHAKE-256s, reflecting differences in the underlying parameterization of the SLH-DSA construction rather than security level alone. In contrast, verification remains substantially less expensive than signing across all parameter sets, although its execution time also increases with the complexity of the selected configuration.

Despite the significant computational overhead, all evaluated SLH-DSA configurations completed successfully on the ESP32-C6 without external memory support. The minimum observed free heap ranged from approximately 204 kB to 246 kB across all workload measurements, indicating that memory consumption remained within the capabilities of the platform. The reduction in available memory compared to the baseline measurements is primarily attributable to the concurrent BLE subsystem and application workload rather than to the cryptographic operations themselves.

For completeness, the minimum largest free heap block was also monitored during all SLH-DSA workload measurements. Observed values ranged from approximately 184 kB to 229 kB depending on the evaluated parameter set. Although the available contiguous memory region decreased due to concurrent system activity, no allocation failures or observable heap fragmentation effects were detected.

Overall, the results indicate that SLH-DSA deployment on the ESP32-C6 is primarily constrained by computational cost rather than memory availability. While all evaluated parameter sets remain technically feasible, the extremely long execution times of SHAKE-based variants substantially limit their practical applicability on resource-constrained embedded platforms, particularly for latency-sensitive applications.

A similar non-monotonic relationship between security category and execution time has also been reported in previous studies using liboqs-based SPHINCS+ implementations, where the 192s variants occasionally exhibited higher execution times than the corresponding 256s configurations.

**Fig 2:**
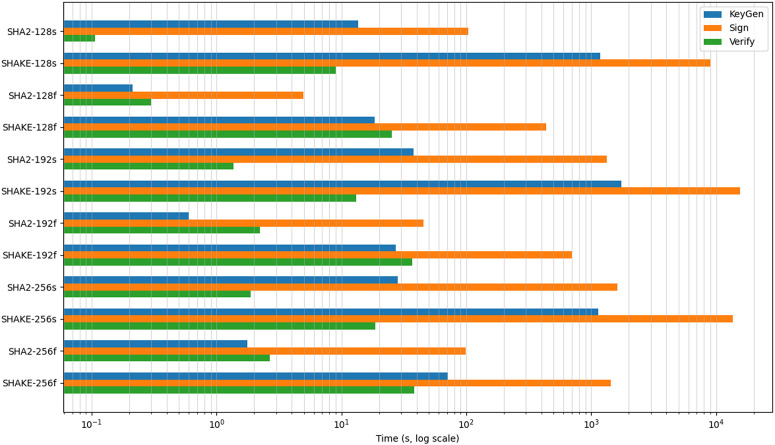
SLH-DSA execution time on ESP32-C6 under a realistic workload (log scale). Key generation, signing, and verification times for SHA2- and SHAKE-based SLH-DSA parameter sets. A logarithmic scale is used to highlight order-of-magnitude differences between the evaluated variants.

Fig 2 highlights the substantial performance gap between SHA2- and SHAKE-based SLH-DSA variants. While SHA2-based parameter sets complete all operations within seconds to tens of minutes, SHAKE-based variants exhibit execution times one to two orders of magnitude higher, with signing dominating overall runtime. This behavior fundamentally limits the practicality of SHAKE-based SLH-DSA on resource-constrained embedded platforms.

### Impact of hardware SHA acceleration

The ESP32-C6 provides dedicated hardware acceleration for SHA-256 and SHA-512 operations, which are extensively used by the SHA2-based SLH-DSA parameter sets. To quantify the impact of hardware-assisted hashing, additional baseline measurements were performed using both software-based and hardware-accelerated SHA implementations.

As shown in Table 12, hardware SHA acceleration provides consistent performance improvements across all evaluated SLH-DSA-SHA2 configurations. The effect is relatively modest for the 128-bit security parameter sets, where speedups remain close to 1.2–1.3×. However, the benefit becomes substantially more pronounced at higher security levels. For the 192-bit and 256-bit parameter sets, signing and verification operations achieve speedups exceeding 5×, while the largest improvement was observed for SLH-DSA-SHA2-256s signing, where hardware acceleration reduced execution time by more than an order of magnitude (10.1×).

**Table 12:** Impact of ESP32-C6 hardware SHA acceleration on SLH-DSA-SHA2 performance. Baseline execution times obtained using software and hardware-accelerated SHA implementations.

Algorithm	Operation	SW SHA (s)	HW SHA (s)	Speedup
SLH-DSA-SHA2-128s	KeyGen	12.33	9.49	1.30×
SLH-DSA-SHA2-128s	Sign	93.67	75.54	1.24×
SLH-DSA-SHA2-128s	Verify	0.088	0.076	1.16×
SLH-DSA-SHA2-128f	KeyGen	0.194	0.156	1.24×
SLH-DSA-SHA2-128f	Sign	4.51	3.64	1.24×
SLH-DSA-SHA2-128f	Verify	0.278	0.228	1.22×
SLH-DSA-SHA2-192s	KeyGen	34.57	15.10	2.29×
SLH-DSA-SHA2-192s	Sign	1252.44	158.73	7.89×
SLH-DSA-SHA2-192s	Verify	1.278	0.136	9.41×
SLH-DSA-SHA2-192f	KeyGen	0.543	0.237	2.29×
SLH-DSA-SHA2-192f	Sign	42.43	6.80	6.24×
SLH-DSA-SHA2-192f	Verify	2.080	0.368	5.65×
SLH-DSA-SHA2-256s	KeyGen	25.97	10.07	2.58×
SLH-DSA-SHA2-256s	Sign	1515.62	149.62	10.13×
SLH-DSA-SHA2-256s	Verify	1.770	0.205	8.62×
SLH-DSA-SHA2-256f	KeyGen	1.625	0.630	2.58×
SLH-DSA-SHA2-256f	Sign	92.59	14.13	6.55×
SLH-DSA-SHA2-256f	Verify	2.493	0.377	6.61×

These results demonstrate that dedicated hash acceleration can significantly improve the practicality of hash-based post-quantum signatures on constrained embedded platforms. Since SHAKE-based SLH-DSA variants rely on Keccak-derived extendable-output functions rather than SHA-2 primitives, they cannot directly benefit from the ESP32-C6 SHA accelerator, which partially explains the substantially higher execution times observed for the SHAKE-based parameter sets.

### Program memory footprint measurement

Program memory footprint was evaluated using the ESP-IDF size analysis tool. For each configuration, the firmware was rebuilt with all workload-related components disabled, allowing the contribution of individual post-quantum cryptographic algorithm families to be assessed in isolation. The reported values correspond to the total firmware image size, including application code, statically linked libraries, and associated metadata. All results are reported relative to a baseline firmware containing the evaluation framework but no PQC algorithm implementations.

The results summarized in Table 13 demonstrate that program memory footprint is strongly dependent on the selected PQC algorithm family. Enabling ML-KEM increases the firmware image size by approximately 91.8 kB (58.8%) relative to the baseline framework, resulting in a total image size of 248 kB. In contrast, both ML-DSA and SLH-DSA introduce substantially larger code footprints, increasing the firmware size by approximately 253 kB and resulting in total image sizes exceeding 409 kB.

**Table 13:** Program memory footprint for evaluated firmware configurations. Total firmware image size and Flash memory increase relative to the baseline framework without PQC support.

Firmware configuration	Image size (B)	Increase (B)	Increase (%)
Baseline framework	156204	–	–
ML-KEM enabled	248012	91808	58.77
ML-DSA enabled	409060	252856	161.87
SLH-DSA (SHA2) enabled	409704	253500	162.29
SLH-DSA (SHAKE) enabled	409536	253332	162.18

Only minor differences were observed between the SHA2- and SHAKE-based SLH-DSA implementations, with the SHA2 configuration requiring 168 B more Flash memory than the SHAKE configuration. This indicates that the dominant memory overhead originates from the shared SLH-DSA implementation framework rather than the selected hash function.

Overall, the results show that all evaluated PQC algorithm families can be integrated within the Flash memory constraints of the ESP32-C6 platform. However, signature schemes impose significantly higher program memory overhead than ML-KEM, which should be considered when deploying post-quantum cryptography on resource-constrained embedded systems.

### Comparison with embedded PQC implementations

Table 14 compares the ESP32-C6 results obtained in this work with representative embedded PQC implementations reported in the literature. The comparison includes Cortex-M4 implementations evaluated within the PQClean and pqm4 frameworks, as well as highly optimized automotive-oriented implementations targeting Infineon TriCore platforms [21].

**Table 14:** Comparison of post-quantum cryptographic implementations across embedded architectures. RISC-V results correspond to the NIST-standardized ML-KEM and ML-DSA implementations evaluated in this work. Cortex-M and TriCore values are derived from literature reporting the corresponding pre-standardized Kyber and Dilithium implementations using PQClean, pqm4, and architecture-specific optimized implementations. Results are intended to provide architectural and implementation-level context rather than a strict like-for-like comparison.

Algorithm	Operation	RISC-V	Cortex-M		TriCore
			PQClean	pqm4	custom opt.
ML-KEM-512	KeyGen	103 ms	32.48 ms	25.71 ms	2.28 ms
ML-KEM-512	Encaps	107 ms	44.24 ms	32.64 ms	2.82 ms
ML-KEM-512	Decaps	129 ms	49.26 ms	31.06 ms	3.08 ms
ML-KEM-768	KeyGen	172 ms	59.83 ms	48.84 ms	3.31 ms
ML-KEM-768	Encaps	171 ms	74.50 ms	57.33 ms	4.01 ms
ML-KEM-768	Decaps	205 ms	80.69 ms	54.74 ms	3.92 ms
ML-KEM-1024	KeyGen	261 ms	94.59 ms	78.75 ms	5.37 ms
ML-KEM-1024	Encaps	264 ms	112.74 ms	88.99 ms	5.95 ms
ML-KEM-1024	Decaps	310 ms	120.39 ms	85.47 ms	5.71 ms
ML-DSA-44	KeyGen	396 ms	—	58.35 ms	—
ML-DSA-44	Sign	843 ms	—	256.54 ms	—
ML-DSA-44	Verify	380 ms	—	60.89 ms	—
ML-DSA-65	KeyGen	717 ms	—	95.10 ms	—
ML-DSA-65	Sign	1318 ms	—	387.06 ms	—
ML-DSA-65	Verify	665 ms	—	92.87 ms	—
ML-DSA-87	KeyGen	1234 ms	—	129.06 ms	—
ML-DSA-87	Sign	1830 ms	—	352.87 ms	—
ML-DSA-87	Verify	1186 ms	—	132.23 ms	—

To facilitate a fair comparison with previously published benchmarks, the RISC-V values reported in Table 14 correspond to the baseline ESP32-C6 measurements obtained without additional application workload, BLE communication, or Wi-Fi activity. This configuration most closely matches the benchmarking methodology used in the referenced studies, which typically evaluate cryptographic primitives in isolation on dedicated evaluation platforms.

The compared studies represent different classes of embedded architectures. ARM Cortex-M4 microcontrollers constitute the most widely investigated platform for embedded PQC evaluation and benefit from mature optimization frameworks such as pqm4. In contrast, the ESP32-C6 represents a low-cost RISC-V IoT platform for which architecture-specific PQC optimizations remain relatively limited. Automotive-oriented TriCore platforms provide substantially higher performance through aggressive implementation optimizations and performance-oriented hardware architectures.

The comparison shows that optimized Cortex-M4 and TriCore implementations generally achieve lower execution times than the portable liboqs-based implementation evaluated on the ESP32-C6. These differences are expected and primarily arise from architecture-specific optimization techniques, including optimized arithmetic routines, memory layouts, and efficient polynomial and NTT implementations. Nevertheless, the ESP32-C6 results remain within the same order of magnitude and demonstrate the practical feasibility of deploying NIST-standardized post-quantum cryptography on resource-constrained RISC-V IoT devices.

It is also important to distinguish between algorithm standardization status and implementation framework. This work evaluates the NIST-standardized algorithms ML-KEM and ML-DSA through the liboqs framework, whereas many embedded benchmarking studies report results for the corresponding pre-standardized algorithm names Kyber and Dilithium. Although these algorithms belong to the same cryptographic families, implementation details and optimization strategies may differ between the evaluated software frameworks.

## Discussion

The presented results provide a comprehensive assessment of the practical feasibility of NIST-standardized post-quantum cryptographic algorithms on a constrained embedded platform. By evaluating ML-KEM, ML-DSA, and SLH-DSA on the same ESP32-C6 microcontroller without external memory support, this study enables a direct comparison of algorithmic trade-offs under realistic deployment constraints.

The ESP32-C6 was intentionally selected as a highly resource-constrained baseline platform, representing a lower bound for practical post-quantum cryptography deployment in IoT-class microcontrollers. Consequently, the measured execution times and memory characteristics should be interpreted as conservative estimates for similar embedded systems.

The combined ML-KEM and ML-DSA evaluation further indicates that complete post-quantum authentication and key-establishment workflows can be executed entirely on the ESP32-C6. Although the combined execution time reaches several seconds for the highest security-level configuration, all evaluated combinations completed successfully within the available memory budget, even under realistic IoT workload conditions.

### Feasibility of lattice-based post-quantum cryptography

The experimental results demonstrate that lattice-based schemes, namely ML-KEM and ML-DSA, are technically feasible on low-cost embedded hardware such as the ESP32-C6. All evaluated parameter sets successfully execute within the available internal memory budget and without requiring external RAM.

The additional experiments conducted under concurrent sensor processing, BLE telemetry, and Wi-Fi connectivity further demonstrated that the evaluated lattice-based schemes remain operational under realistic IoT workloads, with execution-time increases remaining moderate compared to the substantial reduction in available memory caused by wireless subsystem activity.

Among the evaluated algorithms, ML-KEM exhibits the most favorable performance profile. Execution times scale predictably with the security level, and dynamic memory usage remains stable across all parameter sets. These properties make ML-KEM a practical candidate for embedded key exchange, secure channel establishment, and device onboarding protocols in IoT systems.

ML-DSA incurs a higher computational cost, primarily due to expensive signing operations. Nevertheless, its performance remains acceptable for embedded use cases where signatures are generated infrequently, such as device provisioning, firmware authentication, or secure boot verification. In such scenarios, the observed execution times do not pose a fundamental barrier to deployment.

### Hash-based signatures on constrained devices

In contrast to lattice-based schemes, the hash-based SLH-DSA signature scheme presents significant challenges for embedded deployment. While all evaluated SLH-DSA variants successfully execute end-to-end on the ESP32-C6, the associated computational cost is substantially higher, particularly for key generation and signing operations.

For SHA2-based SLH-DSA variants, key generation and signing complete within seconds to minutes, while verification remains comparatively lightweight. In contrast, SHAKE-based variants incur extreme execution times, with signing and verification extending to several hours for higher security categories. These runtimes dominate overall execution cost and severely limit practical deployability on deeply constrained microcontrollers.

As a result, SLH-DSA is not well suited for frequent on-device signing in embedded systems. Its practical use is better restricted to scenarios where signing is performed off-device by a trusted authority, while embedded devices only perform verification. In such hybrid deployment models, SLH-DSA can still provide strong security guarantees without imposing prohibitive computational overhead on the embedded target.

### Security level and performance trade-offs

Across most evaluated algorithms, increasing the NIST security level generally results in higher execution time and, to a lesser extent, increased memory consumption. Exceptions were observed for certain SLH-DSA parameter sets due to differences in algorithm parameterization rather than security level alone. This highlights the importance of careful parameter selection in embedded deployments, where application-level timing constraints and energy consumption are often more critical than absolute cryptographic throughput.

For many IoT applications, lower or intermediate security categories may already provide sufficient post-quantum security while maintaining acceptable performance. At the same time, conservative parameter choices may be justified for long-lived devices exposed to future cryptanalytic advances, including quantum-accelerated lattice reduction techniques. The empirical results presented in this study provide quantitative data to support informed trade-offs between security margin and practical deployability.

### Implications for embedded system design

The findings of this work indicate that post-quantum cryptography can already be integrated into embedded systems based on commodity microcontrollers without external memory support. However, algorithm selection and usage patterns must be carefully adapted to platform constraints.

In addition to runtime and RAM requirements, program memory footprint must also be considered. While all evaluated implementations fit comfortably within the Flash resources of the ESP32-C6, signature schemes introduce substantially larger code footprints than ML-KEM, increasing firmware size by approximately 250 kB relative to the baseline framework.

Lattice-based schemes such as ML-KEM and ML-DSA can be executed fully on-device and offer a favorable balance between security, performance, and memory usage. In contrast, SLH-DSA is primarily constrained by computational cost rather than memory availability, making it more suitable for hybrid designs where signing is performed off-device and verification is performed on the embedded target.

Future embedded platforms equipped with hardware acceleration for polynomial arithmetic or hash-based constructions may significantly reduce the observed performance gap. Until such capabilities become widespread, software-only implementations must carefully balance security requirements, execution time, and energy consumption when deploying post-quantum cryptographic primitives in resource-constrained environments.

The SLH-DSA results further demonstrate the importance of hardware-assisted cryptographic primitives. The ESP32-C6 SHA accelerator substantially reduced execution times for SHA2-based SLH-DSA parameter sets, in some cases by more than an order of magnitude. Future embedded platforms equipped with dedicated accelerators for polynomial arithmetic, SHA-3/Keccak operations, or other post-quantum cryptographic building blocks may further reduce the performance gap observed in this study.

## Conclusion

This work presented an experimental evaluation of NIST-standardized post-quantum cryptographic algorithms on a constrained embedded platform. By benchmarking ML-KEM, ML-DSA, and SLH-DSA on the ESP32-C6 microcontroller without external memory support, this study provides a realistic assessment of execution time, dynamic memory behavior, and practical feasibility for embedded deployments.

The results demonstrate that lattice-based schemes, ML-KEM and ML-DSA, are already deployable on low-cost microcontrollers. ML-KEM exhibits the most favorable performance characteristics, with predictable execution time and stable memory usage across all evaluated security levels, making it well suited for embedded key exchange and secure communication protocols. ML-DSA incurs higher computational cost, particularly for signing operations, but remains practical for embedded use cases where signatures are generated infrequently, such as device provisioning or firmware authentication.

In contrast, SLH-DSA exhibits extreme computational overhead for key generation and signing on the ESP32-C6, which renders it impractical for frequent on-device signing on resource-constrained hardware. Nevertheless, its efficient verification performance and conservative, hash-based security assumptions make SLH-DSA suitable for hybrid deployment models, in which signing is performed off-device and verification is executed on embedded targets.

Although energy consumption was not directly measured in this study, the observed execution times provide an indication of the expected energy cost. In battery-powered IoT devices, energy consumption is closely related to the duration of active processor utilization. Consequently, the extremely long execution times observed for several SLH-DSA parameter sets, particularly the SHAKE-based variants, are expected to translate into substantially higher energy requirements than those associated with lattice-based schemes such as ML-KEM and ML-DSA. While all evaluated configurations were technically feasible on the ESP32-C6, their practical applicability in energy-constrained devices may be limited unless infrequent operation, external power availability, or additional hardware acceleration mechanisms are available.

Furthermore, the combined evaluation of ML-KEM and ML-DSA demonstrated that both mechanisms can be deployed together on the ESP32-C6, enabling complete post-quantum secure communication workflows including key establishment and authentication without requiring external memory resources.

Overall, the findings indicate that post-quantum cryptography can already be integrated into embedded systems using commodity microcontrollers, provided that algorithm selection and usage patterns are carefully adapted to platform constraints. Lattice-based schemes offer a viable balance between security and performance for embedded deployments today, while hash-based signatures remain limited by computational cost rather than memory availability. These results provide empirical guidance for the design of post-quantum–secure embedded systems under realistic resource constraints.

## Future work

Future work will extend the presented study toward a deeper implementation-level analysis of post-quantum cryptography on constrained embedded platforms. In particular, subsequent research will focus on quantifying energy consumption and execution–energy trade-offs for NIST-standardized algorithms across different security levels and parameter sets. Such measurements are essential for assessing the suitability of post-quantum primitives in battery-powered and energy-constrained devices, where computational cost directly translates into operational lifetime.

Another direction of future work involves the investigation of implementation-level side-channel leakage characteristics and attack-surface analysis. This includes experimental evaluation of timing, memory-access, and power-consumption variability, as well as feasibility studies of practical side-channel attack scenarios on software implementations of post-quantum algorithms. The objective is not only to identify potential leakage sources, but also to derive guidelines for constant-time execution and implementation hardening on resource-constrained microcontrollers.

The developed embedded evaluation framework will be further expanded to support systematic optimization experiments, including algorithmic parameter tuning, memory layout adjustments, and compiler-level optimizations. This will enable controlled studies of performance–security trade-offs and facilitate reproducible benchmarking across multiple hardware targets.

## Supporting information

S1 Table NIST-standardized post-quantum algorithms and their parameter sets (FIPS 203–205). Family, parameter set (NIST name), primitive type, and claimed NIST security level.
(PDF)
