# Supplementary material for: Exploring hardware implementation feasibility of post-quantum cryptography in embedded systems: Evaluation of NIST-standardized ML-KEM, ML-DSA and SLH-DSA on ESP32-C6
Source: PLoS One. 2026 Aug 18;21(8):e0355179. doi: 10.1371/journal.pone.0355179 (PMC13485069; doi:10.1371/journal.pone.0355179)
Supplement: Supplementary file 1 [file pone.0355179.s001.pdf]

Supporting information

S1 Table. NIST-standardized post-quantum algorithms and their parameter sets (FIPS 203–205). Family, parameter set (NIST name), primitive type, and claimed NIST security level.

**Family**	**Parameter set**	**Primitive**	**NIST security level**
**Module-lattice KEM (ML-KEM; FIPS 203)**			
ML-KEM	ML-KEM-512	KEM (IND-CCA2)	Level 1 (≈128-bit, AES-128)
ML-KEM	ML-KEM-768	KEM (IND-CCA2)	Level 3 (≈192-bit, AES-192)
ML-KEM	ML-KEM-1024	KEM (IND-CCA2)	Level 5 (≈256-bit, AES-256)
**Module-lattice signatures (ML-DSA; FIPS 204)**			
ML-DSA	ML-DSA-44	Digital signature	Level 2 (≈128-bit, AES-128)
ML-DSA	ML-DSA-65	Digital signature	Level 3 (≈192-bit, AES-192)
ML-DSA	ML-DSA-87	Digital signature	Level 5 (≈256-bit, AES-256)
**Stateless hash-based signatures (SLH-DSA; FIPS 205)**			
SLH-DSA	SLH-DSA-SHA2-128s	Digital signature	Level 1 (≈128-bit, AES-128)
SLH-DSA	SLH-DSA-SHA2-128f	Digital signature	Level 1 (≈128-bit, AES-128)
SLH-DSA	SLH-DSA-SHA2-192s	Digital signature	Level 3 (≈192-bit, AES-192)
SLH-DSA	SLH-DSA-SHA2-192f	Digital signature	Level 3 (≈192-bit, AES-192)
SLH-DSA	SLH-DSA-SHA2-256s	Digital signature	Level 5 (≈256-bit, AES-256)
SLH-DSA	SLH-DSA-SHA2-256f	Digital signature	Level 5 (≈256-bit, AES-256)
SLH-DSA	SLH-DSA-SHAKE-128s	Digital signature	Level 1 (≈128-bit, AES-128)
SLH-DSA	SLH-DSA-SHAKE-128f	Digital signature	Level 1 (≈128-bit, AES-128)
SLH-DSA	SLH-DSA-SHAKE-192s	Digital signature	Level 3 (≈192-bit, AES-192)
SLH-DSA	SLH-DSA-SHAKE-192f	Digital signature	Level 3 (≈192-bit, AES-192)
SLH-DSA	SLH-DSA-SHAKE-256s	Digital signature	Level 5 (≈256-bit, AES-256)
SLH-DSA	SLH-DSA-SHAKE-256f	Digital signature	Level 5 (≈256-bit, AES-256)
