# Supplementary material for: Exploring hardware implementation feasibility of post-quantum cryptography in embedded systems: Evaluation of NIST-standardized ML-KEM, ML-DSA and SLH-DSA on ESP32-C6
Source: PLoS One. 2026 Aug 18;21(8):e0355179. doi: 10.1371/journal.pone.0355179 (PMC13485069; doi:10.1371/journal.pone.0355179)
Supplement: Supplementary file 2 [file pone.0355179.s003.pdf]

Dear Editor and Reviewers,

We would like to sincerely thank the Academic Editor and the Reviewers for their thorough evaluation of our manuscript and for their constructive comments and suggestions. Their feedback helped us improve the quality, clarity, and practical relevance of our work.

In response to the comments, we have carefully revised the manuscript and introduced several substantial improvements. In particular, we extended the experimental evaluation to include realistic embedded workload scenarios, analyzed the impact of wireless subsystems, added program memory footprint measurements, and incorporated combined cryptographic operation evaluations. We also strengthened the statistical rigor of the SLH-DSA measurements and expanded the reproducibility details, including implementation and configuration parameters.

Furthermore, we addressed the concerns related to data availability by ensuring that all relevant experimental data and supporting materials are clearly documented and accessible. Minor issues related to formatting, presentation, and typographical consistency have also been corrected throughout the manuscript.

All changes are clearly indicated in the revised manuscript with track changes. Below, we provide a detailed, point-by-point response to all comments raised by the Academic Editor and the Reviewers.

2. Journal Requirements

When submitting your revision, we need you to address these additional requirements.

2.1 Formatting

Please ensure that your manuscript meets PLOS ONE's style requirements, including those for file naming. The PLOS ONE style templates can be found at

https://journals.plos.org/plosone/s/file?id=wjVg/PLOSOne_formatting_sample_main_body.pdf and https://journals.plos.org/plosone/s/file?id=ba62/PLOSOne_formatting_sample_title_authors_affiliations.pdf

Response:

The manuscript has been updated to comply with PLOS ONE’s style requirements.

2.2 Funding statement

Thank you for stating the following in the Acknowledgments Section of your manuscript:
"This work was supported by the EU Horizon Europe Framework Program under Grant Agreement no. 101225759 (PQ-NEXT: Post-Quantum Networks for Energy-eXfficient Transitions)."

We note that you have provided funding information that is not currently declared in your Funding Statement. However, funding information should not appear in the Acknowledgments section or other areas of your manuscript. We will only publish funding information present in the Funding Statement section of the online submission form.
Please remove any funding-related text from the manuscript and let us know how you would like to update your Funding Statement. Currently, your Funding Statement reads as follows:
"This work was supported by the EU Horizon Europe Framework Programme under Grant Agreement No. 101225759 (PQ-NEXT: Post-Quantum Networks for Energy-eXfficient Transitions). The funders had no role in study design, data collection and analysis, decision to publish, or preparation of the manuscript."

Please include your amended statements within your cover letter; we will change the online submission form on your behalf.

Response:

We thank the Editor for this clarification. The funding information has been removed from the Acknowledgments section of the manuscript to comply with PLOS ONE requirements. The Funding Statement has been retained and is provided in the submission system. Minor typographical inconsistencies in the funding description have also been corrected from "Energy-eXfficient Transitions" to "Energy-Efficient Transitions".

2.3 Supporting information

We notice that your supplementary tables are included in the manuscript file. Please remove them and upload them with the file type 'Supporting Information'. Please ensure that each Supporting Information file has a legend listed in the manuscript after the references list.

Response:

Thank you for this observation. Following the recommendation, all supplementary tables have been removed from the main manuscript and uploaded separately as Supporting Information files. In addition, appropriate Supporting Information legends have been added to the manuscript after the references section, in accordance with the journal guidelines.

2.4 Citation recommendations

If the reviewer comments include a recommendation to cite specific previously published works, please review and evaluate these publications to determine whether they are relevant and should be cited. There is no requirement to cite these works unless the editor has indicated otherwise.

Response:

We reviewed the references suggested during the review process and evaluated their relevance to the present study. Any necessary changes to the reference list were made in the revised manuscript.

3. Additional Editor Comments

This manuscript presents a timely and useful experimental study of NIST-standardized post-quantum cryptography on the ESP32-C6 platform. The reviewers recognize its practical relevance, clear writing, and valuable open-source contribution. However, the current version requires major revision to improve experimental realism, methodological rigor, and practical depth. In particular, the manuscript should better address realistic embedded workloads, wireless subsystem effects, program memory footprint, combined deployment of KEM and signatures, and the role of hardware acceleration. The statistical treatment of SLH-DSA measurements and the reproducibility details should also be strengthened. Several minor presentation and typographical issues also require correction.

Response:

We thank the Academic Editor for the detailed assessment and constructive recommendations. In response, we substantially revised the manuscript to address all major concerns. Specifically, we extended the experimental evaluation to include realistic embedded workload scenarios, investigated the impact of active wireless subsystems, added program memory footprint measurements, and incorporated evaluations of combined cryptographic operations representative of practical deployments. We also expanded the discussion of hardware acceleration by evaluating the impact of the ESP32-C6 hardware SHA accelerator on selected cryptographic workloads and

discussing its implications for post-quantum cryptography in embedded systems. Furthermore, the statistical treatment of SLH-DSA measurements was strengthened through additional analysis and repeated measurements, while the reproducibility of the study was improved by providing more detailed implementation, configuration, and experimental setup information. Finally, all identified presentation, formatting, and typographical issues were carefully corrected throughout the revised manuscript.

4. Reviewer #1

The manuscript entitled "Exploring Hardware Implementation Feasibility of Post-Quantum Cryptography in Embedded Systems: Evaluation of NIST-Standardized ML-KEM, ML-DSA and SLH-DSA on ESP32-C6" presents an experimental evaluation of NIST-standardized post-quantum cryptographic primitives on a resource-constrained embedded platform. The work evaluates three major PQC primitives (ML-KEM, ML-DSA, and SLH-DSA) using an ESP32-C6 microcontroller and provides measurements on execution latency and memory usage.

The topic is timely and relevant to the ongoing transition toward post-quantum cryptography, particularly for IoT and embedded devices. The study provides useful engineering-level insights into the feasibility of deploying standardized PQC primitives on low-cost microcontrollers.

However, from the perspective of a high-impact information security journal, the current manuscript still lacks several important aspects related to realistic deployment conditions, experimental completeness, performance analysis depth, and statistical rigor. In particular, the experimental setup reflects an idealized cryptographic benchmark environment rather than a realistic embedded system workload.

Therefore, major revisions are required before the manuscript can be considered for publication.

The following major and minor comments are provided to help the authors improve the technical depth and practical relevance of the work.

4.1 Major Comment 1

Lack of Realistic Embedded Workload Scenarios

The current evaluation measures cryptographic performance in isolation. In contrast, in practical MCU-based systems, cryptographic operations rarely execute as standalone workloads and are typically integrated with application-level tasks such as sensor data processing, communication stack operations, real-time task scheduling, and wireless networking. Consequently, evaluating cryptographic primitives in a pure benchmark environment may not accurately reflect real-world deployment conditions. The authors are therefore encouraged to extend their experiments by introducing representative application workloads, such as periodic sensor acquisition tasks, background data processing, or communication stack operations,

during cryptographic execution. Such additional experiments would enable a more comprehensive assessment of how cryptographic operations interact with typical application workloads, thereby providing a more realistic evaluation of the feasibility of PQC deployment on resource-constrained embedded systems.

Response:

We thank the Reviewer for this valuable suggestion. We agree that evaluating cryptographic algorithms in isolation does not fully capture the conditions encountered in practical embedded deployments. In response, we substantially extended the experimental evaluation by introducing representative embedded workload scenarios executed concurrently with PQC operations. Specifically, we evaluated the impact of periodic sensor acquisition and processing tasks, as well as active wireless communication subsystems (Wi-Fi and Bluetooth Low Energy), on the execution characteristics of the evaluated algorithms. These additional experiments provide a more realistic assessment of PQC deployment on resource-constrained embedded platforms and allow us to analyze the interaction between cryptographic operations and typical embedded system workloads. The methodology, results, and discussion of these experiments have been added to the revised manuscript.

4.2 Major Comment 2

Impact of Wireless Subsystems on Cryptographic Performance

The ESP32-C6 microcontroller is widely used in wireless IoT devices where Wi-Fi and Bluetooth connectivity are frequently enabled, yet the manuscript does not clarify whether these wireless subsystems were enabled or disabled during benchmarking. This represents a significant limitation because wireless drivers and RF subsystems may introduce CPU scheduling interference, increased memory pressure, and interrupt overhead. Therefore, the authors should clarify the experimental configuration and, if possible, provide additional measurements under realistic operating conditions where Wi-Fi is enabled, Bluetooth is enabled, or both subsystems operate concurrently. Including such results would significantly strengthen the practical relevance of the study by demonstrating the impact of wireless communication on cryptographic performance.

Response:

We thank the Reviewer for this important observation. In the original version of the manuscript, all benchmark measurements were performed with wireless subsystems disabled in order to isolate the computational characteristics of the evaluated cryptographic algorithms. To address the Reviewer's concern and improve the practical relevance of the study, we extended the experimental evaluation to include additional measurements with Wi-Fi enabled, Bluetooth Low Energy (BLE) enabled, These experiments allow us to assess the impact of wireless communication activities, including scheduling overhead, interrupt handling, and resource contention, on the performance of the evaluated PQC algorithms. The experimental methodology, results, and corresponding discussion have been added to the revised manuscript.

4.3 Major Comment 3

<u>*Limited Comparison with Other Embedded PQC Implementations*</u>
The current evaluation relies solely on liboqs-based implementations of the PQC algorithms, which provide portable reference implementations but do not account for specialized embedded PQC frameworks such as pqm4, PQClean, and ARM-optimized implementations for Cortex-M microcontrollers. These specialized frameworks include critical architecture-specific optimizations, including assembly-level arithmetic optimizations, memory layout optimizations, and specialized NTT implementations. To improve the completeness of the evaluation, the authors are encouraged to include comparisons with optimized implementations from these frameworks or, at a minimum, provide a detailed discussion regarding the expected performance differences between portable implementations and optimized embedded implementations. Furthermore, comparing performance across different MCU architectures, such as ARM Cortex-M versus RISC-V, would significantly strengthen the overall contribution and breadth of the study.

<u>Response:</u>

Thank you for this valuable suggestion. To address this comment, we added a new subsection entitled "Comparison with embedded PQC implementations" and included Table 15, which compares the ESP32-C6 results obtained in this work with representative embedded implementations reported in the literature. The comparison now includes PQClean implementations, optimized Cortex-M4 implementations from the pqm4 framework, and highly optimized Infineon TriCore implementations.

We also expanded the discussion regarding differences between portable liboqs implementations and architecture-specific embedded implementations. The revised manuscript explains that pqm4 and other optimized frameworks benefit from assembly-level arithmetic optimizations, optimized

memory layouts, and efficient NTT implementations, whereas the ESP32-C6 evaluation intentionally uses portable liboqs implementations to provide reproducible cross-platform measurements.

In addition, we included a comparison across different embedded architectures (RISC-V, ARM Cortex-M, and TriCore), thereby providing broader architectural context for the reported results. The manuscript explicitly notes that these comparisons should be interpreted as contextual architectural comparisons rather than strict implementation-equivalence benchmarks because the referenced studies use different hardware platforms, software frameworks, and benchmarking methodologies.

4.4 Major Comment 4

Program Memory Footprint Analysis

While the manuscript reports runtime memory behavior such as heap usage, it does not evaluate the program memory footprint of the PQC implementations, which is a critical factor in embedded systems where available flash memory is often strictly limited. Integrating cryptographic libraries into real-world applications may significantly increase the overall firmware size, potentially exceeding the storage capacity of constrained IoT devices. The authors should therefore provide specific measurements for the firmware size increase caused by each PQC primitive, the total code size of the cryptographic library, and the combined size when integrated with a representative application. This detailed analysis would provide valuable insights into the practical feasibility of integrating PQC libraries into constrained IoT firmware and assist developers in making informed resource allocation decisions.

Response:

Thank you for this valuable suggestion. To address this comment, we added a new subsection entitled “Program memory footprint measurement” together with a dedicated table reporting firmware image size measurements obtained using the ESP-IDF size analysis tools.

The revised manuscript now reports the baseline firmware size, the incremental image size associated with each evaluated PQC family (ML-KEM, ML-DSA, and SLH-DSA). For each configuration, both the absolute increase in image size and the relative percentage increase are provided.

Unlike runtime heap measurements reported elsewhere in the manuscript, these results quantify the impact of PQC integration on non-volatile program memory consumption, which is a critical

resource in embedded systems. The additional measurements demonstrate that all evaluated PQC configurations remain deployable on the ESP32-C6 platform while providing practical insight into the flash-memory overhead associated with integrating post-quantum cryptographic functionality into realistic IoT firmware.

4.5 Major Comment 5

Combined Use of KEM and Signature Algorithms

In most practical security protocols used in IoT systems, such as TLS and device authentication frameworks, cryptographic primitives are used in combination and typically involve both key encapsulation mechanisms like ML-KEM and digital signature algorithms like ML-DSA. While the current study evaluates each primitive independently, it does not analyze the combined system cost, which is essential for understanding real-world performance. The authors are therefore encouraged to evaluate representative combinations, such as ML-KEM-512 with ML-DSA-44 or ML-KEM-768 with ML-DSA-65, and report the combined execution latency and memory usage. Such an analysis would better reflect real-world cryptographic deployment scenarios and provide a more accurate assessment of how these algorithms perform when integrated into standard security protocols.

Response:

Thank you for this valuable suggestion. To address this comment, we added a dedicated subsection entitled "Combined ML-KEM and ML-DSA evaluation" together with Table 8.

The revised manuscript now evaluates representative combinations of NIST-standardized key encapsulation and digital signature algorithms, namely ML-KEM-512 with ML-DSA-44, ML-KEM-768 with ML-DSA-65, and ML-KEM-1024 with ML-DSA-87. Each benchmark includes sequential execution of key generation, encapsulation, decapsulation, signing, and verification operations within a single measurement window.

In addition to total execution time, the revised evaluation reports memory availability metrics, including minimum free heap and minimum largest free heap block. Measurements are provided both for baseline conditions and for a realistic IoT workload scenario including sensor acquisition, background processing, BLE communication, and Wi-Fi connectivity.

These additional experiments provide a protocol-oriented view of PQC deployment and demonstrate the practical feasibility of combined ML-KEM and ML-DSA operation on the ESP32-C6 platform.

4.6 Major Comment 6

Use of Hardware Cryptographic Accelerators

According to the ESP32-C6 hardware specification, the microcontroller includes dedicated hardware acceleration for SHA operations, which is particularly relevant since SLH-DSA relies heavily on intensive hash computations. It is therefore important to clarify whether the hardware SHA accelerator was utilized during the evaluation to ensure the performance data is accurately contextualized. The authors should explicitly state whether hardware hash acceleration was enabled, identify which specific cryptographic primitives took advantage of the accelerator, and explain how the use of such hardware affected the overall performance. In the event that hardware acceleration was not employed, the authors should provide a clear justification for this design choice and discuss its implications for the study's conclusions regarding the efficiency of these post-quantum algorithms on the ESP32-C6 platform.

Response:

Thank you for this important observation. The revised manuscript now explicitly describes the use of hardware cryptographic acceleration available on the ESP32-C6 platform.

All evaluated PQC implementations utilized the ESP32-C6 hardware true random number generator (TRNG) through the underlying ESP-IDF and liboqs software stack whenever cryptographically secure randomness was required. Consequently, operations such as key generation and signature generation benefited from hardware-assisted entropy generation rather than software-based pseudo-random number generation.

In addition, we extended the evaluation by investigating the impact of the ESP32-C6 hardware SHA accelerator on SHA2-based SLH-DSA implementations. A dedicated benchmark table was added to compare software-based and hardware-accelerated SHA execution. The results show measurable performance improvements across all evaluated SHA2-based SLH-DSA parameter sets, with speedups ranging from approximately 1.2× to more than 10× depending on the operation and security level.

The manuscript also clarifies that SHAKE-based SLH-DSA variants cannot directly utilize the ESP32-C6 SHA accelerator because they rely on Keccak-derived extendable-output functions rather than SHA-2 primitives.

These additions provide a more complete characterization of the impact of available hardware cryptographic acceleration on PQC performance for the ESP32-C6 platform.

4.7 Major Comment 7

<u>Statistical Validity of SLH-DSA Measurements</u>
Due to the long execution time associated with SLH-DSA operations, the manuscript reports single-run measurements for certain configurations, which is understandable from a practical standpoint but remains statistically weak. Single measurements may be significantly affected by external factors such as system warm-up effects, cache behavior, temperature variations, and the current memory allocation state. The authors should therefore attempt to repeat these measurements multiple times, providing at least several runs where feasible, and report comprehensive statistical metrics including the mean, standard deviation, and minimum and maximum values. Implementing such a multi-run approach would significantly improve the reliability and reproducibility of the reported results, ensuring that the performance evaluation of SLH-DSA is both robust and scientifically sound.

<u>Response:</u>

Thank you for this valuable suggestion. We agree that single-run measurements are insufficient for robust statistical evaluation, particularly for computationally intensive cryptographic algorithms.

To address this concern, we redesigned the SLH-DSA benchmarking methodology in the revised manuscript. The original single-run measurements were replaced with repeated measurements and statistical reporting. For all SHA2-based SLH-DSA parameter sets, results are now reported as mean ± standard deviation over 10 independent runs. For the significantly more computationally demanding SHAKE-based parameter sets, measurements were repeated 3 times and are likewise reported as mean ± standard deviation.

The revised benchmark tables therefore provide statistically representative execution-time measurements rather than single observations. The number of repetitions for SHAKE-based variants was limited by their extremely long execution times, with some configurations requiring several hours per benchmark run. Nevertheless, multiple independent measurements were collected for all evaluated configurations.

These changes substantially improve the reliability, reproducibility, and statistical validity of the reported SLH-DSA performance results.

4.8 Minor Comment 1

<u>Table 11 presentation</u>
Table 11 currently mixes milliseconds and seconds in the same comparison table, which may confuse readers. The authors should standardize the units or clearly separate the measurements.

<u>Response:</u>

Thank you for this observation. We agree that presenting milliseconds and seconds within the same comparison table could potentially lead to confusion.

In the revised manuscript, the table in question has been removed because its contents are now covered by the more comprehensive comparison presented in Table 14 As a result, the issue of mixed time units no longer occurs in the revised version of the manuscript.

4.9 Minor Comment 2

<u>Reference ordering</u>
The order of references in the bibliography appears inconsistent in several places. The authors should ensure that references are correctly ordered and formatted according to the journal guidelines.

<u>Response:</u>

Thank you for pointing this out. The bibliography and all in-text citations have been carefully reviewed in the revised manuscript. Reference entries were verified, corrected where necessary, and reordered to ensure consistency with the citation order and compliance with the journal formatting requirements. We also reviewed the completeness and formatting of the bibliographic records to improve overall consistency and readability.

4.10 Minor Comment 3

<u>Experimental reproducibility</u>
The manuscript would significantly benefit from the inclusion of more detailed experimental information to improve the overall reproducibility of the study. Specifically, the authors should provide the exact version of the liboqs library used, the specific compiler version, along with the optimization flags applied during the build process, and the version of the ESP-IDF framework

utilized for the ESP32-C6 platform. Providing these technical details is essential for allowing other researchers to accurately replicate the experiments and verify the reported performance metrics across similar embedded environments.

Response:

Thank you for this suggestion. We expanded the Software stack subsection to improve experimental reproducibility. The revised manuscript now explicitly reports the ESP-IDF version, liboqs version, compiler toolchain version, and the build optimization configuration used during the experiments.

Specifically, the firmware was built using ESP-IDF v5.5.1, liboqs v0.14.0, and riscv32-esp-elf-gcc GCC 14.2.0 with the ESP-IDF default debug optimization profile. These details were added to allow other researchers to reproduce the reported measurements under the same software and build environment.

5. Reviewer #2

The manuscript presents a timely, highly practical, and well-executed evaluation of NIST-standardized post-quantum cryptographic algorithms on a resource-constrained microcontroller. By targeting a device without external RAM (the ESP32-C6) and successfully benchmarking ML-KEM, ML-DSA, and SLH-DSA end-to-end, the authors provide valuable empirical data for the embedded systems and IoT communities.

The paper is well-written, the methodology is sound, and the open-source release of the evaluation framework is highly commendable. I recommend this manuscript for publication following a revision to address the following technical clarifications and typographical issues.

5.1 Comment 1

The manuscript states that due to extreme runtimes, SLH-DSA variants were measured using single executions. While this is entirely understandable for the SHAKE-based variants that take hours to compute, some of the SHA2-based "fast" variants complete in fractions of a second (e.g., SLH-DSA-SHA2-128f takes 0.19s for KeyGen and 4.51s for signing). The authors should consider running the faster SLH-DSA variants multiple times (e.g., 100 runs, as done for ML-KEM and ML-DSA) to provide a standard deviation and ensure methodological consistency where practically feasible.

Response:

Thank you for this valuable suggestion. We agree that repeated measurements are important for ensuring statistical validity and methodological consistency.

In the revised manuscript, the SLH-DSA evaluation was substantially expanded. The previous single-run measurements were replaced with repeated benchmark campaigns and statistical reporting. For the SHA2-based variants, all reported values now correspond to the mean and standard deviation obtained from 10 independent runs. For the significantly more computationally intensive SHAKE-based variants, measurements were repeated 3 times and the mean and standard deviation are reported.

These changes provide a more robust characterization of execution-time variability while remaining practically feasible given the extremely long runtimes of the higher-security SHAKE-based parameter sets.

5.2 Comment 2

The authors correctly monitored the largest free heap block and observed no observable heap fragmentation effects across the algorithms. It would be beneficial to add a brief sentence explaining why this is the case (e.g., clarifying if the memory allocation and deallocation patterns within the configured liboqs are strictly sequential).

Response:

Thank you for this valuable observation. We agree that additional context regarding the absence of observable heap fragmentation improves the interpretation of the reported memory measurements.

In the revised manuscript, we added a short explanation to the Results section clarifying that, in addition to monitoring the minimum available heap memory, the largest free heap block was monitored throughout all benchmark campaigns as an indicator of heap fragmentation. No observable fragmentation effects were detected for any evaluated algorithm. We further explain that this behavior is likely related to the memory-management characteristics of the evaluated liboqs implementations, which primarily allocate temporary buffers during cryptographic operations and release them upon completion. Consequently, long-term heap fragmentation effects remain limited despite the substantial differences in execution time between the evaluated parameter sets.

5.3 Comment 3

The authors note the use of the ESP-IDF esp_random() interface, sourcing entropy exclusively from the hardware TRNG. The authors should briefly clarify in the text whether pulling large amounts of entropy continuously from the TRNG introduces any significant blocking latency during key generation or signing, compared to using a deterministic random bit generator (DRBG) seeded by the TRNG.

Response:

Thank you for this valuable observation. We added a clarification regarding the source of randomness used during the experiments and its potential impact on performance.

The evaluated implementation obtains randomness directly from the ESP32-C6 hardware TRNG through the ESP-IDF esp_random() interface via a custom liboqs RNG callback. No software-based pseudo-random number generator or deterministic random bit generator (DRBG) was used in the evaluation framework. We further clarified that, although random number generation is required during key generation and certain signing operations, its contribution to the overall execution time is expected to be negligible compared to the dominant computational costs of the evaluated PQC algorithms, including polynomial arithmetic, hashing, and signature processing. Consequently, the direct use of the hardware TRNG is not expected to significantly influence the reported benchmark results.

This clarification has been added to the Software stack subsection of the revised manuscript.

5.4 Comment 4

The comparative baseline provided against x86-64 CPUs successfully highlights order-of-magnitude differences. However, as this is an embedded systems paper, briefly contrasting the ESP32-C6 results with other common embedded architectures mentioned in the literature review (such as ARM Cortex-M4 platforms) would help readers contextualize the 32-bit RISC-V performance relative to its direct peers.

Response:

Thank you for this valuable suggestion. We agree that comparisons with other embedded architectures provide important context for interpreting the ESP32-C6 results.

In the revised manuscript, the previous CPU-oriented comparison was replaced by a dedicated subsection entitled "Comparison with embedded PQC implementations" together with a new comparative table. The revised comparison now includes representative results reported for ARM Cortex-M4 platforms through the PQClean and pqm4 frameworks, as well as highly optimized automotive-oriented implementations targeting Infineon TriCore architectures.

In addition to the quantitative comparison, we added a discussion describing the architectural differences between Cortex-M4, RISC-V, and TriCore platforms, the impact of architecture-specific optimizations, and the limitations associated with direct cross-platform comparisons. These additions provide readers with substantially improved context for evaluating the performance of the ESP32-C6 relative to its embedded-system peers.

5.5 Comment 5

The authors mention in the "Future Work" section that they plan to evaluate energy consumption. Given the extreme execution times observed for SLH-DSA (up to several hours), adding a brief qualitative statement in the Discussion section regarding how these runtimes theoretically impact the energy budget of battery-powered IoT devices would strengthen the paper's practical conclusions.

Response:

Thank you for this valuable suggestion. We agree that the practical implications of the observed execution times should be discussed from the perspective of energy-constrained embedded systems.

In the revised manuscript, we added a qualitative discussion to the Conclusion section addressing the expected impact of execution time on the energy budget of battery-powered IoT devices. While energy consumption was not directly measured in this study, we note that the extremely long execution times observed for several SLH-DSA parameter sets, particularly the SHAKE-based variants, are expected to result in substantially higher energy requirements due to prolonged processor activity. We further clarify that, although all evaluated configurations were successfully

executed on the ESP32-C6, their practical deployment in energy-constrained devices may require infrequent operation, external power availability, or future hardware acceleration mechanisms.

This addition provides practical deployment context while remaining consistent with the scope of the present work.

5.6 Comment 6

In both the Financial Disclosure and the Acknowledgments sections, the grant title contains a typo: "Energy-eXfficient Transitions". This should be corrected to "Energy-Efficient Transitions" prior to publication.

Response:

We thank the reviewer for identifying this typographical error. The grant title has been corrected from “Energy-eXfficient Transitions” to “Energy-Efficient Transitions”. In addition, in accordance with PLOS ONE requirements, funding information has been removed from the Acknowledgments section and is now provided exclusively in the Funding Statement.
