# Supplementary material for: Exploring hardware implementation feasibility of post-quantum cryptography in embedded systems: Evaluation of NIST-standardized ML-KEM, ML-DSA and SLH-DSA on ESP32-C6
Source: PLoS One. 2026 Aug 18;21(8):e0355179. doi: 10.1371/journal.pone.0355179 (PMC13485069; doi:10.1371/journal.pone.0355179)
Supplement: Peer review history

# Peer review: Decision Letter 0

**DOI:** 10.1371/journal.pone.0355179.r001

**Authors:** Chun-Wei Yang

15 Mar 2026

PONE-D-26-08044

Exploring hardware implementation feasibility of post-quantum cryptography in embedded systems: Evaluation of NIST-standardized ML-KEM, ML-DSA and SLH-DSA on ESP32-C6

PLOS One

Dear Dr. Karcz,

Thank you for submitting your manuscript to PLOS ONE. After careful consideration, we feel that it has merit but does not fully meet PLOS ONE’s publication criteria as it currently stands. Therefore, we invite you to submit a revised version of the manuscript that addresses the points raised during the review process.

Please submit your revised manuscript by Apr 29 2026 11:59PM. If you will need more time than this to complete your revisions, please reply to this message or contact the journal office at plosone@plos.org. When you're ready to submit your revision, log on to https://www.editorialmanager.com/pone/ and select the 'Submissions Needing Revision' folder to locate your manuscript file.

Please include the following items when submitting your revised manuscript:

- A letter that responds to each point raised by the academic editor and reviewer(s). You should upload this letter as a separate file labeled 'Response to Reviewers'.
- A marked-up copy of your manuscript that highlights changes made to the original version. You should upload this as a separate file labeled 'Revised Manuscript with Track Changes'.
- An unmarked version of your revised paper without tracked changes. You should upload this as a separate file labeled 'Manuscript'.If you would like to make changes to your financial disclosure, please include your updated statement in your cover letter. Guidelines for resubmitting your figure files are available below the reviewer comments at the end of this letter.

If applicable, we recommend that you deposit your laboratory protocols in protocols.io to enhance the reproducibility of your results. Protocols.io assigns your protocol its own identifier (DOI) so that it can be cited independently in the future. For instructions see: https://journals.plos.org/plosone/s/submission-guidelines#loc-laboratory-protocols. Additionally, PLOS ONE offers an option for publishing peer-reviewed Lab Protocol articles, which describe protocols hosted on protocols.io. Read more information on sharing protocols at https://plos.org/protocols?utm_medium=editorial-email&utm_source=authorletters&utm_campaign=protocols.

We look forward to receiving your revised manuscript.

Kind regards,

Chun-Wei Yang, Ph.D.

Academic Editor

PLOS One

Journal Requirements:

When submitting your revision, we need you to address these additional requirements.

1. Please ensure that your manuscript meets PLOS ONE's style requirements, including those for file naming. The PLOS ONE style templates can be found at

https://journals.plos.org/plosone/s/file?id=wjVg/PLOSOne_formatting_sample_main_body.pdf and

https://journals.plos.org/plosone/s/file?id=ba62/PLOSOne_formatting_sample_title_authors_affiliations.pdf

2.  Thank you for stating the following in the Acknowledgments Section of your manuscript:

“This work was supported by the EU Horizon Europe Framework Program under Grant Agreement no. 101225759 (PQ-NEXT: Post-Quantum Networks for Energy-eXfficient Transitions).”

We note that you have provided funding information that is not currently declared in your Funding Statement. However, funding information should not appear in the Acknowledgments section or other areas of your manuscript. We will only publish funding information present in the Funding Statement section of the online submission form.

Please remove any funding-related text from the manuscript and let us know how you would like to update your Funding Statement. Currently, your Funding Statement reads as follows:

“This work was supported by the EU Horizon Europe Framework Programme under Grant Agreement No. 101225759 (PQ-NEXT: Post-Quantum Networks for Energy-eXfficient Transitions). The funders had no role in study design, data collection and analysis, decision to publish, or preparation of the manuscript.”

Please include your amended statements within your cover letter; we will change the online submission form on your behalf.

3. We notice that your supplementary tables are included in the manuscript file. Please remove them and upload them with the file type 'Supporting Information'. Please ensure that each Supporting Information file has a legend listed in the manuscript after the references list.

4. If the reviewer comments include a recommendation to cite specific previously published works, please review and evaluate these publications to determine whether they are relevant and should be cited. There is no requirement to cite these works unless the editor has indicated otherwise.

Additional Editor Comments:

This manuscript presents a timely and useful experimental study of NIST-standardized post-quantum cryptography on the ESP32-C6 platform. The reviewers recognize its practical relevance, clear writing, and valuable open-source contribution. However, the current version requires major revision to improve experimental realism, methodological rigor, and practical depth. In particular, the manuscript should better address realistic embedded workloads, wireless subsystem effects, program memory footprint, combined deployment of KEM and signatures, and the role of hardware acceleration. The statistical treatment of SLH-DSA measurements and the reproducibility details should also be strengthened. Several minor presentation and typographical issues also require correction.

Reviewers' comments:

Reviewer's Responses to Questions

**Comments to the Author**

1. Is the manuscript technically sound, and do the data support the conclusions?

The manuscript must describe a technically sound piece of scientific research with data that supports the conclusions. Experiments must have been conducted rigorously, with appropriate controls, replication, and sample sizes. The conclusions must be drawn appropriately based on the data presented.

Reviewer #1: Yes

Reviewer #2: Yes

**********

2. Has the statistical analysis been performed appropriately and rigorously?

Reviewer #1: No

Reviewer #2: Yes

**********

3. Have the authors made all data underlying the findings in their manuscript fully available?

The PLOS Data policy requires authors to make all data underlying the findings described in their manuscript fully available without restriction, with rare exception (please refer to the Data Availability Statement in the manuscript PDF file). The data should be provided as part of the manuscript or its supporting information, or deposited to a public repository. For example, in addition to summary statistics, the data points behind means, medians and variance measures should be available. If there are restrictions on publicly sharing data—e.g. participant privacy or use of data from a third party—those must be specified.

Reviewer #1: No

Reviewer #2: Yes

**********

4. Is the manuscript presented in an intelligible fashion and written in standard English?

PLOS ONE does not copyedit accepted manuscripts, so the language in submitted articles must be clear, correct, and unambiguous. Any typographical or grammatical errors should be corrected at revision, so please note any specific errors here.

Reviewer #1: Yes

Reviewer #2: Yes

**********

5. Review Comments to the Author

Please use the space provided to explain your answers to the questions above. You may also include additional comments for the author, including concerns about dual publication, research ethics, or publication ethics. (Please upload your review as an attachment if it exceeds 20,000 characters)

Reviewer #1: The manuscript entitled “Exploring Hardware Implementation Feasibility of Post-Quantum Cryptography in Embedded Systems: Evaluation of NIST-Standardized ML-KEM, ML-DSA and SLH-DSA on ESP32-C6” presents an experimental evaluation of NIST-standardized post-quantum cryptographic primitives on a resource-constrained embedded platform. The work evaluates three major PQC primitives (ML-KEM, ML-DSA, and SLH-DSA) using an ESP32-C6 microcontroller and provides measurements on execution latency and memory usage.

The topic is timely and relevant to the ongoing transition toward post-quantum cryptography, particularly for IoT and embedded devices. The study provides useful engineering-level insights into the feasibility of deploying standardized PQC primitives on low-cost microcontrollers.

However, from the perspective of a high-impact information security journal, the current manuscript still lacks several important aspects related to realistic deployment conditions, experimental completeness, performance analysis depth, and statistical rigor. In particular, the experimental setup reflects an idealized cryptographic benchmark environment rather than a realistic embedded system workload.

Therefore, major revisions are required before the manuscript can be considered for publication.

The following major and minor comments are provided to help the authors improve the technical depth and practical relevance of the work.

Major Comments

1. Lack of Realistic Embedded Workload Scenarios

The current evaluation measures cryptographic performance in isolation. In contrast, in practical MCU-based systems, cryptographic operations rarely execute as standalone workloads and are typically integrated with application-level tasks such as sensor data processing, communication stack operations, real-time task scheduling, and wireless networking. Consequently, evaluating cryptographic primitives in a pure benchmark environment may not accurately reflect real-world deployment conditions. The authors are therefore encouraged to extend their experiments by introducing representative application workloads, such as periodic sensor acquisition tasks, background data processing, or communication stack operations, during cryptographic execution. Such additional experiments would enable a more comprehensive assessment of how cryptographic operations interact with typical application workloads, thereby providing a more realistic evaluation of the feasibility of PQC deployment on resource-constrained embedded systems.

2. Impact of Wireless Subsystems on Cryptographic Performance

The ESP32-C6 microcontroller is widely used in wireless IoT devices where Wi-Fi and Bluetooth connectivity are frequently enabled, yet the manuscript does not clarify whether these wireless subsystems were enabled or disabled during benchmarking. This represents a significant limitation because wireless drivers and RF subsystems may introduce CPU scheduling interference, increased memory pressure, and interrupt overhead. Therefore, the authors should clarify the experimental configuration and, if possible, provide additional measurements under realistic operating conditions where Wi-Fi is enabled, Bluetooth is enabled, or both subsystems operate concurrently. Including such results would significantly strengthen the practical relevance of the study by demonstrating the impact of wireless communication on cryptographic performance.

3. Limited Comparison with Other Embedded PQC Implementations

The current evaluation relies solely on liboqs-based implementations of the PQC algorithms, which provide portable reference implementations but do not account for specialized embedded PQC frameworks such as pqm4, PQClean, and ARM-optimized implementations for Cortex-M microcontrollers. These specialized frameworks include critical architecture-specific optimizations, including assembly-level arithmetic optimizations, memory layout optimizations, and specialized NTT implementations. To improve the completeness of the evaluation, the authors are encouraged to include comparisons with optimized implementations from these frameworks or, at a minimum, provide a detailed discussion regarding the expected performance differences between portable implementations and optimized embedded implementations. Furthermore, comparing performance across different MCU architectures, such as ARM Cortex-M versus RISC-V, would significantly strengthen the overall contribution and breadth of the study.

4. Program Memory Footprint Analysis

While the manuscript reports runtime memory behavior such as heap usage, it does not evaluate the program memory footprint of the PQC implementations, which is a critical factor in embedded systems where available flash memory is often strictly limited. Integrating cryptographic libraries into real-world applications may significantly increase the overall firmware size, potentially exceeding the storage capacity of constrained IoT devices. The authors should therefore provide specific measurements for the firmware size increase caused by each PQC primitive, the total code size of the cryptographic library, and the combined size when integrated with a representative application. This detailed analysis would provide valuable insights into the practical feasibility of integrating PQC libraries into constrained IoT firmware and assist developers in making informed resource allocation decisions.

5. Combined Use of KEM and Signature Algorithms

In most practical security protocols used in IoT systems, such as TLS and device authentication frameworks, cryptographic primitives are used in combination and typically involve both key encapsulation mechanisms like ML-KEM and digital signature algorithms like ML-DSA. While the current study evaluates each primitive independently, it does not analyze the combined system cost, which is essential for understanding real-world performance. The authors are therefore encouraged to evaluate representative combinations, such as ML-KEM-512 with ML-DSA-44 or ML-KEM-768 with ML-DSA-65, and report the combined execution latency and memory usage. Such an analysis would better reflect real-world cryptographic deployment scenarios and provide a more accurate assessment of how these algorithms perform when integrated into standard security protocols.

6. Use of Hardware Cryptographic Accelerators

According to the ESP32-C6 hardware specification, the microcontroller includes dedicated hardware acceleration for SHA operations, which is particularly relevant since SLH-DSA relies heavily on intensive hash computations. It is therefore important to clarify whether the hardware SHA accelerator was utilized during the evaluation to ensure the performance data is accurately contextualized. The authors should explicitly state whether hardware hash acceleration was enabled, identify which specific cryptographic primitives took advantage of the accelerator, and explain how the use of such hardware affected the overall performance. In the event that hardware acceleration was not employed, the authors should provide a clear justification for this design choice and discuss its implications for the study's conclusions regarding the efficiency of these post-quantum algorithms on the ESP32-C6 platform.

7. Statistical Validity of SLH-DSA Measurements

Due to the long execution time associated with SLH-DSA operations, the manuscript reports single-run measurements for certain configurations, which is understandable from a practical standpoint but remains statistically weak. Single measurements may be significantly affected by external factors such as system warm-up effects, cache behavior, temperature variations, and the current memory allocation state. The authors should therefore attempt to repeat these measurements multiple times, providing at least several runs where feasible, and report comprehensive statistical metrics including the mean, standard deviation, and minimum and maximum values. Implementing such a multi-run approach would significantly improve the reliability and reproducibility of the reported results, ensuring that the performance evaluation of SLH-DSA is both robust and scientifically sound.

Minor Comments

1. Table 11 presentation

Table 11 currently mixes milliseconds and seconds in the same comparison table, which may confuse readers. The authors should standardize the units or clearly separate the measurements.

2. Reference ordering

The order of references in the bibliography appears inconsistent in several places. The authors should ensure that references are correctly ordered and formatted according to the journal guidelines.

3. Experimental reproducibility

The manuscript would significantly benefit from the inclusion of more detailed experimental information to improve the overall reproducibility of the study. Specifically, the authors should provide the exact version of the liboqs library used, the specific compiler version, along with the optimization flags applied during the build process, and the version of the ESP-IDF framework utilized for the ESP32-C6 platform. Providing these technical details is essential for allowing other researchers to accurately replicate the experiments and verify the reported performance metrics across similar embedded environments.

Reviewer #2: The manuscript presents a timely, highly practical, and well-executed evaluation of NIST-standardized post-quantum cryptographic algorithms on a resource-constrained microcontroller. By targeting a device without external RAM (the ESP32-C6) and successfully benchmarking ML-KEM, ML-DSA, and SLH-DSA end-to-end, the authors provide valuable empirical data for the embedded systems and IoT communities.

The paper is well-written, the methodology is sound, and the open-source release of the evaluation framework is highly commendable. I recommend this manuscript for publication following a revision to address the following technical clarifications and typographical issues.

1. The manuscript states that due to extreme runtimes, SLH-DSA variants were measured using single executions. While this is entirely understandable for the SHAKE-based variants that take hours to compute, some of the SHA2-based "fast" variants complete in fractions of a second (e.g., SLH-DSA-SHA2-128f takes 0.19s for KeyGen and 4.51s for signing). The authors should consider running the faster SLH-DSA variants multiple times (e.g., 100 runs, as done for ML-KEM and ML-DSA) to provide a standard deviation and ensure methodological consistency where practically feasible.

2. The authors correctly monitored the largest free heap block and observed no observable heap fragmentation effects across the algorithms. It would be beneficial to add a brief sentence explaining why this is the case (e.g., clarifying if the memory allocation and deallocation patterns within the configured liboqs are strictly sequential).

3. The authors note the use of the ESP-IDF esp_random() interface, sourcing entropy exclusively from the hardware TRNG. The authors should briefly clarify in the text whether pulling large amounts of entropy continuously from the TRNG introduces any significant blocking latency during key generation or signing, compared to using a deterministic random bit generator (DRBG) seeded by the TRNG.

4. The comparative baseline provided against x86-64 CPUs successfully highlights order-of-magnitude differences. However, as this is an embedded systems paper, briefly contrasting the ESP32-C6 results with other common embedded architectures mentioned in the literature review (such as ARM Cortex-M4 platforms) would help readers contextualize the 32-bit RISC-V performance relative to its direct peers.

5. The authors mention in the "Future Work" section that they plan to evaluate energy consumption. Given the extreme execution times observed for SLH-DSA (up to several hours), adding a brief qualitative statement in the Discussion section regarding how these runtimes theoretically impact the energy budget of battery-powered IoT devices would strengthen the paper's practical conclusions.

6. In both the Financial Disclosure and the Acknowledgments sections, the grant title contains a typo: "Energy-eXfficient Transitions". This should be corrected to "Energy-Efficient Transitions" prior to publication.

**********

6. PLOS authors have the option to publish the peer review history of their article (what does this mean?). If published, this will include your full peer review and any attached files.

If you choose “no”, your identity will remain anonymous but your review may still be made public.

**Do you want your identity to be public for this peer review?**  For information about this choice, including consent withdrawal, please see our Privacy Policy.

Reviewer #1: No

Reviewer #2: No

**********

[NOTE: If reviewer comments were submitted as an attachment file, they will be attached to this email and accessible via the submission site. Please log into your account, locate the manuscript record, and check for the action link "View Attachments". If this link does not appear, there are no attachment files.]

To ensure your figures meet our technical requirements, please review our figure guidelines: https://journals.plos.org/plosone/s/figures

You may also use PLOS’s free figure tool, NAAS, to help you prepare publication quality figures: https://journals.plos.org/plosone/s/figures#loc-tools-for-figure-preparation.

NAAS will assess whether your figures meet our technical requirements by comparing each figure against our figure specifications.

# Author comment: Author response to Decision Letter 1

**DOI:** 10.1371/journal.pone.0355179.r002

12 Jun 2026

Dear Editor and Reviewers,

We would like to sincerely thank the Academic Editor and the Reviewers for their thorough evaluation of our manuscript and for their constructive comments and suggestions. Their feedback helped us improve the quality, clarity, and practical relevance of our work.

In response to the comments, we have carefully revised the manuscript and introduced several substantial improvements. In particular, we extended the experimental evaluation to include realistic embedded workload scenarios, analyzed the impact of wireless subsystems, added program memory footprint measurements, and incorporated combined cryptographic operation evaluations. We also strengthened the statistical rigor of the SLH-DSA measurements and expanded the reproducibility details, including implementation and configuration parameters.

Furthermore, we addressed the concerns related to data availability by ensuring that all relevant experimental data and supporting materials are clearly documented and accessible. Minor issues related to formatting, presentation, and typographical consistency have also been corrected throughout the manuscript.

All changes are clearly indicated in the revised manuscript with track changes. Below, we provide a detailed, point-by-point response to all comments raised by the Academic Editor and the Reviewers.

2. Journal Requirements

When submitting your revision, we need you to address these additional requirements.

2.1 Formatting

Please ensure that your manuscript meets PLOS ONE's style requirements, including those for file naming. The PLOS ONE style templates can be found at

https://journals.plos.org/plosone/s/file?id=wjVg/PLOSOne_formatting_sample_main_body.pdf and https://journals.plos.org/plosone/s/file?id=ba62/PLOSOne_formatting_sample_title_authors_affiliations.pdf

Response:

The manuscript has been updated to comply with PLOS ONE’s style requirements.

2.2 Funding statement

Thank you for stating the following in the Acknowledgments Section of your manuscript:

“This work was supported by the EU Horizon Europe Framework Program under Grant Agreement no. 101225759 (PQ-NEXT: Post-Quantum Networks for Energy-eXfficient Transitions).”

We note that you have provided funding information that is not currently declared in your Funding Statement. However, funding information should not appear in the Acknowledgments section or other areas of your manuscript. We will only publish funding information present in the Funding Statement section of the online submission form.

Please remove any funding-related text from the manuscript and let us know how you would like to update your Funding Statement. Currently, your Funding Statement reads as follows:

“This work was supported by the EU Horizon Europe Framework Programme under Grant Agreement No. 101225759 (PQ-NEXT: Post-Quantum Networks for Energy-eXfficient Transitions). The funders had no role in study design, data collection and analysis, decision to publish, or preparation of the manuscript.”

Please include your amended statements within your cover letter; we will change the online submission form on your behalf.

Response:

We thank the Editor for this clarification. The funding information has been removed from the Acknowledgments section of the manuscript to comply with PLOS ONE requirements. The Funding Statement has been retained and is provided in the submission system. Minor typographical inconsistencies in the funding description have also been corrected from “Energy-eXfficient Transitions” to “Energy-Efficient Transitions”.

2.3 Supporting information

We notice that your supplementary tables are included in the manuscript file. Please remove them and upload them with the file type 'Supporting Information'. Please ensure that each Supporting Information file has a legend listed in the manuscript after the references list.

Response:

Thank you for this observation. Following the recommendation, all supplementary tables have been removed from the main manuscript and uploaded separately as Supporting Information files. In addition, appropriate Supporting Information legends have been added to the manuscript after the references section, in accordance with the journal guidelines.

2.4 Citation recommendations

If the reviewer comments include a recommendation to cite specific previously published works, please review and evaluate these publications to determine whether they are relevant and should be cited. There is no requirement to cite these works unless the editor has indicated otherwise.

Response:

We reviewed the references suggested during the review process and evaluated their relevance to the present study. Any necessary changes to the reference list were made in the revised manuscript.

3. Additional Editor Comments

This manuscript presents a timely and useful experimental study of NIST-standardized post-quantum cryptography on the ESP32-C6 platform. The reviewers recognize its practical relevance, clear writing, and valuable open-source contribution. However, the current version requires major revision to improve experimental realism, methodological rigor, and practical depth. In particular, the manuscript should better address realistic embedded workloads, wireless subsystem effects, program memory footprint, combined deployment of KEM and signatures, and the role of hardware acceleration. The statistical treatment of SLH-DSA measurements and the reproducibility details should also be strengthened. Several minor presentation and typographical issues also require correction.

Response:

We thank the Academic Editor for the detailed assessment and constructive recommendations. In response, we substantially revised the manuscript to address all major concerns. Specifically, we extended the experimental evaluation to include realistic embedded workload scenarios, investigated the impact of active wireless subsystems, added program memory footprint measurements, and incorporated evaluations of combined cryptographic operations representative of practical deployments. We also expanded the discussion of hardware acceleration by evaluating the impact of the ESP32-C6 hardware SHA accelerator on selected cryptographic workloads and discussing its implications for post-quantum cryptography in embedded systems. Furthermore, the statistical treatment of SLH-DSA measurements was strengthened through additional analysis and repeated measurements, while the reproducibility of the study was improved by providing more detailed implementation, configuration, and experimental setup information. Finally, all identified presentation, formatting, and typographical issues were carefully corrected throughout the revised manuscript.

4. Reviewer #1

The manuscript entitled “Exploring Hardware Implementation Feasibility of Post-Quantum Cryptography in Embedded Systems: Evaluation of NIST-Standardized ML-KEM, ML-DSA and SLH-DSA on ESP32-C6” presents an experimental evaluation of NIST-standardized post-quantum cryptographic primitives on a resource-constrained embedded platform. The work evaluates three major PQC primitives (ML-KEM, ML-DSA, and SLH-DSA) using an ESP32-C6 microcontroller and provides measurements on execution latency and memory usage.

The topic is timely and relevant to the ongoing transition toward post-quantum cryptography, particularly for IoT and embedded devices. The study provides useful engineering-level insights into the feasibility of deploying standardized PQC primitives on low-cost microcontrollers.

However, from the perspective of a high-impact information security journal, the current manuscript still lacks several important aspects related to realistic deployment conditions, experimental completeness, performance analysis depth, and statistical rigor. In particular, the experimental setup reflects an idealized cryptographic benchmark environment rather than a realistic embedded system workload.

Therefore, major revisions are required before the manuscript can be considered for publication.

The following major and minor comments are provided to help the authors improve the technical depth and practical relevance of the work.

4.1 Major Comment 1

Lack of Realistic Embedded Workload Scenarios

The current evaluation measures cryptographic performance in isolation. In contrast, in practical MCU-based systems, cryptographic operations rarely execute as standalone workloads and are typically integrated with application-level tasks such as sensor data processing, communication stack operations, real-time task scheduling, and wireless networking. Consequently, evaluating cryptographic primitives in a pure benchmark environment may not accurately reflect real-world deployment conditions. The authors are therefore encouraged to extend their experiments by introducing representative application workloads, such as periodic sensor acquisition tasks, background data processing, or communication stack operations, during cryptographic execution. Such additional experiments would enable a more comprehensive assessment of how cryptographic operations interact with typical application workloads, thereby providing a more realistic evaluation of the feasibility of PQC deployment on resource-constrained embedded systems.

Response:

We thank the Reviewer for this valuable suggestion. We agree that evaluating cryptographic algorithms in isolation does not fully capture the conditions encountered in practical embedded deployments. In response, we substantially extended the experimental evaluation by introducing representative embedded workload scenarios executed concurrently with PQC operations. Specifically, we evaluated the impact of periodic sensor acquisition and processing tasks, as well as active wireless communication subsystems (Wi-Fi and Bluetooth Low Energy), on the execution characteristics of the evaluated algorithms. These additional experiments provide a more realistic assessment of PQC deployment on resource-constrained embedded platforms and allow us to analyze the interaction between cryptographic operations and typical embedded system workloads. The methodology, results, and discussion of these experiments have been added to the revised manuscript.

4.2 Major Comment 2

Impact of Wireless Subsystems on Cryptographic Performance

The ESP32-C6 microcontroller is widely used in wireless IoT devices where Wi-Fi and Bluetooth connectivity are frequently enabled, yet the manuscript does not clarify whether these wireless subsystems were enabled or disabled during benchmarking. This represents a significant limitation because wireless drivers and RF subsystems may introduce CPU scheduling interference, increased memory pressure, and interrupt overhead. Therefore, the authors should clarify the experimental configuration and, if possible, provide additional measurements under realistic operating conditions where Wi-Fi is enabled, Bluetooth is enabled, or both subsystems operate concurrently. Including such results would significantly strengthen the practical relevance of the study by demonstrating the impact of wireless communication on cryptographic performance.

Response:

We thank the Reviewer for this important observation. In the original version of the manuscript, all benchmark measurements were performed with wireless subsystems disabled in order to isolate the computational characteristics of the evaluated cryptographic algorithms. To address the Reviewer's concern and improve the practical relevance of the study, we extended the experimental evaluation to include additional measurements with Wi-Fi enabled, Bluetooth Low Energy (BLE) enabled, These experiments allow us to assess the impact of wireless communication activities, including scheduling overhead, interrupt handling, and resource contention, on the performance of the evaluated PQC algorithms. The experimental methodology, results, and corresponding discussion have been added to the revised manuscript.

4.3 Major Comment 3

Limited Comparison with Other Embedded PQC Implementations

The current evaluation relies solely on liboqs-based implementations of the PQC algorithms, which provide portable reference implementations but do not account for specialized embedded PQC frameworks such as pqm4, PQClean, and ARM-optimized implementations for Cortex-M microcontrollers. These specialized frameworks include critical architecture-specific optimizations, including assembly-level arithmetic optimizations, memory layout optimizations, and specialized NTT implementations. To improve the completeness of the evaluation, the authors are encouraged to include comparisons with optimized implementations from these frameworks or, at a minimum, provide a detailed discussion regarding the expected performance differences between portable implementations and optimized embedded implementations. Furthermore, comparing performance across different MCU architectures, such as ARM Cortex-M versus RISC-V, would significantly strengthen the overall contribution and breadth of the study.

Response:

Thank you for this valuable suggestion. To address this comment, we added a new subsection entitled “Comparison with embedded PQC implementations” and included Table 15, which compares the ESP32-C6 results obtained in this work with representative embedded implementations reported in the literature. The comparison now includes PQClean implementations, optimized Cortex-M4 implementations from the pqm4 framework, and highly optimized Infineon TriCore implementations.

We also expanded the discussion regarding differences between portable liboqs implementations and architecture-specific embedded implementations. The revised manuscript explains that pqm4 and other optimized frameworks benefit from assembly-level arithmetic optimizations, optimized memory layouts, and efficient NTT implementations, whereas the ESP32-C6 evaluation intentionally uses portable liboqs implementations to provide reproducible cross-platform measurements.

In addition, we included a comparison across different embedded architectures (RISC-V, ARM Cortex-M, and TriCore), thereby providing broader architectural context for the reported results. The manuscript explicitly notes that these comparisons should be interpreted as contextual architectural comparisons rather than strict implementation-equivalence benchmarks because the referenced studies use different hardware platforms, software frameworks, and benchmarking methodologies.

4.4 Major Comment 4

Program Memory Footprint Analysis

While the manuscript reports runtime memory behavior such as heap usage, it does not evaluate the program memory footprint of the PQC implementations, which is a critical factor in embedded systems where available flash memory is often strictly limited. Integrating cryptographic libraries into real-world applications may significantly increase the overall firmware size, potentially exceeding the storage capacity of constrained IoT devices. The authors should therefore provide specific measurements for the firmware size increase caused by each PQC primitive, the total code size of the cryptographic library, and the combined size when integrated with a representative application. This detailed analysis would provide valuable insights into the practical feasibility of integrating PQC libraries into constrained IoT firmware and assist developers in making informed resource allocation decisions.

Response:

Thank you for this valuable suggestion. To address this comment, we added a new subsection entitled “Program memory footprint measurement” together with a dedicated table reporting firmware image size measurements obtained using the ESP-IDF size analysis tools.

The revised manuscript now reports the baseline firmware size, the incremental image size associated with each evaluated PQC family (ML-KEM, ML-DSA, and SLH-DSA). For each configuration, both the absolute increase in image size and the relative percentage increase are provided.

Unlike runtime heap measurements reported elsewhere in the manuscript, these results quantify the impact of PQC integration on non-volatile program memory consumption, which is a critical resource in embedded systems. The additional measurements demonstrate that all evaluated PQC c

Attachment Submitted filename: Response to Reviewers.pdf

# Peer review: Decision Letter 1

**DOI:** 10.1371/journal.pone.0355179.r003

**Authors:** Chun-Wei Yang

30 Jun 2026

PONE-D-26-08044R1

Exploring hardware implementation feasibility of post-quantum cryptography in embedded systems: Evaluation of NIST-standardized ML-KEM, ML-DSA and SLH-DSA on ESP32-C6

PLOS One

Dear Dr. Karcz,

Thank you for submitting your manuscript to PLOS ONE. After careful consideration, we feel that it has merit but does not fully meet PLOS ONE’s publication criteria as it currently stands. Therefore, we invite you to submit a revised version of the manuscript that addresses the points raised during the review process.

Please submit your revised manuscript by Aug 14 2026 11:59PM. If you will need more time than this to complete your revisions, please reply to this message or contact the journal office at plosone@plos.org. When you're ready to submit your revision, log on to https://www.editorialmanager.com/pone/ and select the 'Submissions Needing Revision' folder to locate your manuscript file.

Please include the following items when submitting your revised manuscript:

- A letter that responds to each point raised by the academic editor and reviewer(s). You should upload this letter as a separate file labeled 'Response to Reviewers'.
- A marked-up copy of your manuscript that highlights changes made to the original version. You should upload this as a separate file labeled 'Revised Manuscript with Track Changes'.
- An unmarked version of your revised paper without tracked changes. You should upload this as a separate file labeled 'Manuscript'.

If you would like to make changes to your financial disclosure, please include your updated statement in your cover letter. Guidelines for resubmitting your figure files are available below the reviewer comments at the end of this letter.

If applicable, we recommend that you deposit your laboratory protocols in protocols.io to enhance the reproducibility of your results. Protocols.io assigns your protocol its own identifier (DOI) so that it can be cited independently in the future. For instructions see: https://journals.plos.org/plosone/s/submission-guidelines#loc-laboratory-protocols. Additionally, PLOS ONE offers an option for publishing peer-reviewed Lab Protocol articles, which describe protocols hosted on protocols.io. Read more information on sharing protocols at https://plos.org/protocols?utm_medium=editorial-email&utm_source=authorletters&utm_campaign=protocols.

As the corresponding author, your ORCID iD is verified in the submission system and will appear in the published article. PLOS supports the use of ORCID, and we encourage all coauthors to register for an ORCID iD and use it as well. Please encourage your coauthors to verify their ORCID iD within the submission system before final acceptance, as unverified ORCID iDs will not appear in the published article. *Only*  the individual author can complete the verification step; PLOS staff *cannot*  verify ORCID iDs on behalf of authors.

We look forward to receiving your revised manuscript.

Kind regards,

Chun-Wei Yang, Ph.D.

Academic Editor

PLOS One

Journal Requirements:

If the reviewer comments include a recommendation to cite specific previously published works, please review and evaluate these publications to determine whether they are relevant and should be cited. There is no requirement to cite these works unless the editor has indicated otherwise.

Please review your reference list to ensure that it is complete and correct. If you have cited papers that have been retracted, please include the rationale for doing so in the manuscript text, or remove these references and replace them with relevant current references. Any changes to the reference list should be mentioned in the rebuttal letter that accompanies your revised manuscript. If you need to cite a retracted article, indicate the article’s retracted status in the References list and also include a citation and full reference for the retraction notice.

Additional Editor Comments:

The manuscript has been substantially improved, and the reviewers recognize the authors’ diligent efforts in addressing the previous comments. The additional experiments, especially those involving real-world IoT workloads with active Wi-Fi and BLE, as well as the combined ML-KEM/ML-DSA evaluation, significantly enhance the practical relevance of the study. Before final acceptance, please address several minor presentation issues. Clearly indicate where each revision was made in the response letter, correct the table numbering from Table 15 to Table 14 where applicable, fix the incomplete “Figure ??” reference on Line 582, standardize numerical formatting such as “303,104 B,” and conduct a final consistency proofread.

Reviewers' comments:

Reviewer's Responses to Questions

**Comments to the Author**

1. If the authors have adequately addressed your comments raised in a previous round of review and you feel that this manuscript is now acceptable for publication, you may indicate that here to bypass the “Comments to the Author” section, enter your conflict of interest statement in the “Confidential to Editor” section, and submit your "Accept" recommendation.

Reviewer #1: All comments have been addressed

Reviewer #2: All comments have been addressed

**********

2. Is the manuscript technically sound, and do the data support the conclusions?

The manuscript must describe a technically sound piece of scientific research with data that supports the conclusions. Experiments must have been conducted rigorously, with appropriate controls, replication, and sample sizes. The conclusions must be drawn appropriately based on the data presented.

Reviewer #1: Yes

Reviewer #2: Yes

**********

3. Has the statistical analysis been performed appropriately and rigorously?

Reviewer #1: Yes

Reviewer #2: Yes

**********

4. Have the authors made all data underlying the findings in their manuscript fully available?

The PLOS Data policy requires authors to make all data underlying the findings described in their manuscript fully available without restriction, with rare exception (please refer to the Data Availability Statement in the manuscript PDF file). The data should be provided as part of the manuscript or its supporting information, or deposited to a public repository. For example, in addition to summary statistics, the data points behind means, medians and variance measures should be available. If there are restrictions on publicly sharing data—e.g. participant privacy or use of data from a third party—those must be specified.

Reviewer #1: Yes

Reviewer #2: Yes

**********

5. Is the manuscript presented in an intelligible fashion and written in standard English?

PLOS ONE does not copyedit accepted manuscripts, so the language in submitted articles must be clear, correct, and unambiguous. Any typographical or grammatical errors should be corrected at revision, so please note any specific errors here.

Reviewer #1: Yes

Reviewer #2: Yes

**********

6. Review Comments to the Author

Please use the space provided to explain your answers to the questions above. You may also include additional comments for the author, including concerns about dual publication, research ethics, or publication ethics. (Please upload your review as an attachment if it exceeds 20,000 characters)

Reviewer #1: First, I would like to commend you on your diligent and thorough efforts in revising the manuscript. You have clearly addressed each of the reviewer's comments, and the inclusion of additional experimental data has significantly enhanced the paper's value. Your work now stands as a comprehensive guide for the practical implementation of PQC on microcontrollers and is nearing readiness for publication.

Before you prepare the final version, I would like to offer the following suggestions to ensure the manuscript is polished to the highest standard:

1. Mapping Revisions: In your response to the reviewers, it is a best practice to specify where in the manuscript each revision has been made (e.g., "This point is addressed in Section 3.2, Paragraph 4"). This allows editors and reviewers to efficiently verify the changes and appreciate the full context of your updates.

2. Table Numbering Correction: In your response, you mentioned the addition of "Table 15" under the section Comparison with embedded PQC implementation. Please be advised that the correct in the manuscript is Table 14.

3. Missing Figure Reference: The reference on Line 582 is incomplete ("Figure ??"). Please correct this to ensure it points to the intended figure.

4. Numerical Formatting: A formatting inconsistency exists for numerical values. For example, the value on Lines 400-402 is written as "303 104 B", which differs from the format used elsewhere (e.g., "303,104 B"). Please correct this specific instance.

5. Final Proofread for Consistency: Building on the point above, I strongly recommend that you conduct one final, meticulous proofread of the entire manuscript. The goal is to unify the formatting of all text, figures, tables, and numerical values to ensure a professional and consistent presentation throughout the document.

Reviewer #2: The authors have done an excellent job addressing the previous rounds of feedback. The revised manuscript is a major improvement.

The new experiments add a lot of practical value to the study. In particular, testing the algorithms under real-world IoT workloads (with active Wi-Fi and BLE) and evaluating the combined ML-KEM/ML-DSA performance makes the findings much more relevant to practitioners. Therefore, I recommend accepting it in its current form.

**********

7. PLOS authors have the option to publish the peer review history of their article (what does this mean?). If published, this will include your full peer review and any attached files.

If you choose “no”, your identity will remain anonymous but your review may still be made public.

**Do you want your identity to be public for this peer review?**  For information about this choice, including consent withdrawal, please see our Privacy Policy.

Reviewer #1: No

Reviewer #2: No

**********

[NOTE: If reviewer comments were submitted as an attachment file, they will be attached to this email and accessible via the submission site. Please log into your account, locate the manuscript record, and check for the action link "View Attachments". If this link does not appear, there are no attachment files.]

To ensure your figures meet our technical requirements, please review our figure guidelines: https://journals.plos.org/plosone/s/figures

You may also use PLOS’s free figure tool, NAAS, to help you prepare publication quality figures: https://journals.plos.org/plosone/s/figures#loc-tools-for-figure-preparation.

NAAS will assess whether your figures meet our technical requirements by comparing each figure against our figure specifications.

# Author comment: Author response to Decision Letter 2

**DOI:** 10.1371/journal.pone.0355179.r004

3 Jul 2026

Dear Editor and Reviewers,

We sincerely thank the Academic Editor and both Reviewers for their careful evaluation of our revised manuscript. We greatly appreciate the positive assessment of our work and are pleased that the additional experiments and revisions have addressed the previous concerns. We have carefully considered all remaining comments and have revised the manuscript accordingly. All changes are highlighted in the tracked-changes version of the manuscript.

1. Journal Requirements

If the reviewer comments include a recommendation to cite specific previously published works, please review and evaluate these publications to determine whether they are relevant and should be cited. There is no requirement to cite these works unless the editor has indicated otherwise.

Please review your reference list to ensure that it is complete and correct. If you have cited papers that have been retracted, please include the rationale for doing so in the manuscript text, or remove these references and replace them with relevant current references. Any changes to the reference list should be mentioned in the rebuttal letter that accompanies your revised manuscript. If you need to cite a retracted article, indicate the article’s retracted status in the References list and also include a citation and full reference for the retraction notice.

Additional Editor Comments:

The manuscript has been substantially improved, and the reviewers recognize the authors’ diligent efforts in addressing the previous comments. The additional experiments, especially those involving real-world IoT workloads with active Wi-Fi and BLE, as well as the combined ML-KEM/ML-DSA evaluation, significantly enhance the practical relevance of the study. Before final acceptance, please address several minor presentation issues. Clearly indicate where each revision was made in the response letter, correct the table numbering from Table 15 to Table 14 where applicable, fix the incomplete “Figure ??” reference on Line 582, standardize numerical formatting such as “303,104 B,” and conduct a final consistency proofread.

Response: We sincerely thank the Academic Editor for the positive assessment of our revised manuscript and for the constructive suggestions. In response, we have:

• clearly indicated the location of each revision throughout this response letter,

• verified the table numbering and corrected all references where necessary,

• reviewed and confirmed that all figure references are complete and consistent, including the previously incomplete reference to Figure 2,

• standardized the formatting of numerical values throughout the manuscript (e.g., 303,104 B),

• performed a thorough final proofread to ensure consistency in formatting, terminology, figures, tables, and references across the entire manuscript.

2. Review Comments to the Author

Reviewer #1: First, I would like to commend you on your diligent and thorough efforts in revising the manuscript. You have clearly addressed each of the reviewer's comments, and the inclusion of additional experimental data has significantly enhanced the paper's value. Your work now stands as a comprehensive guide for the practical implementation of PQC on microcontrollers and is nearing readiness for publication.

Before you prepare the final version, I would like to offer the following suggestions to ensure the manuscript is polished to the highest standard:

2.1. Mapping Revisions: In your response to the reviewers, it is a best practice to specify where in the manuscript each revision has been made (e.g., "This point is addressed in Section 3.2, Paragraph 4"). This allows editors and reviewers to efficiently verify the changes and appreciate the full context of your updates.

Response: Thank you for this helpful suggestion. In this revised response letter, we explicitly indicate the location of each revision by providing the corresponding section, paragraph, table, or figure reference where applicable.

2.2. Table Numbering Correction: In your response, you mentioned the addition of "Table 15" under the section Comparison with embedded PQC implementation. Please be advised that the correct in the manuscript is Table 14.

Response: Thank you for pointing this out. We verified the table numbering and confirmed that all references in the revised manuscript correctly refer to Table 14.

2.3. Missing Figure Reference: The reference on Line 582 is incomplete ("Figure ??"). Please correct this to ensure it points to the intended figure.

Response: Thank you for pointing this out. We carefully reviewed the SLH-DSA performance section and confirmed that the figure reference has been corrected. The manuscript now consistently refers to Figure 2, and no incomplete figure references remain.

2.4. Numerical Formatting: A formatting inconsistency exists for numerical values. For example, the value on Lines 400-402 is written as "303 104 B", which differs from the format used elsewhere (e.g., "303,104 B"). Please correct this specific instance.

Response: Thank you for pointing out this formatting inconsistency. In accordance with the reviewer’s suggestion, the numerical formatting in the ML-KEM performance section has been corrected. Specifically, the values have been updated to 303,104 B, 229,376 B, and 104,448 B to ensure consistent formatting throughout the manuscript.

2.5. Final Proofread for Consistency: Building on the point above, I strongly recommend that you conduct one final, meticulous proofread of the entire manuscript. The goal is to unify the formatting of all text, figures, tables, and numerical values to ensure a professional and consistent presentation throughout the document.

Response: Thank you for this recommendation. We conducted a thorough final proofread of the entire manuscript to ensure consistency in formatting, terminology, figures, tables, and numerical values. In addition, the complete reference list was carefully reviewed and verified for accuracy, completeness, and formatting consistency. Minor typographical and formatting inconsistencies identified during this review were corrected to ensure a uniform and professional presentation throughout the manuscript.

Reviewer #2: The authors have done an excellent job addressing the previous rounds of feedback. The revised manuscript is a major improvement.

The new experiments add a lot of practical value to the study. In particular, testing the algorithms under real-world IoT workloads (with active Wi-Fi and BLE) and evaluating the combined ML-KEM/ML-DSA performance makes the findings much more relevant to practitioners. Therefore, I recommend accepting it in its current form.

Response: We sincerely thank the reviewer for the positive evaluation of our work and for recognizing the improvements made during the revision process. We appreciate the reviewer’s recommendation for acceptance.

# Peer review: Decision Letter 2

**DOI:** 10.1371/journal.pone.0355179.r005

**Authors:** Chun-Wei Yang

20 Jul 2026

Exploring hardware implementation feasibility of post-quantum cryptography in embedded systems: Evaluation of NIST-standardized ML-KEM, ML-DSA and SLH-DSA on ESP32-C6

PONE-D-26-08044R2

Dear Dr. Karcz,

We’re pleased to inform you that your manuscript has been judged scientifically suitable for publication and will be formally accepted for publication once it meets all outstanding technical requirements.

Within one week, you’ll receive an e-mail detailing the required amendments. When these have been addressed, you’ll receive a formal acceptance letter and your manuscript will be scheduled for publication.

An invoice will be generated when your article is formally accepted. Please note, if your institution has a publishing partnership with PLOS and your article meets the relevant criteria, all or part of your publication costs will be covered. Please make sure your user information is up-to-date by logging into Editorial Manager at Editorial Manager® and clicking the ‘Update My Information' link at the top of the page. For questions related to billing, please contact billing support.

If your institution or institutions have a press office, please notify them about your upcoming paper to help maximize its impact. If they’ll be preparing press materials, please inform our press team as soon as possible -- no later than 48 hours after receiving the formal acceptance. Your manuscript will remain under strict press embargo until 2 pm Eastern Time on the date of publication. For more information, please contact onepress@plos.org.

Kind regards,

Chun-Wei Yang, Ph.D.

Academic Editor

PLOS One

Additional Editor Comments (optional):

The authors have revised the manuscript in accordance with the suggestions from both reviewers; therefore, I recommend acceptance of this paper.

Reviewers' comments:

Reviewer's Responses to Questions

**Comments to the Author**

1. If the authors have adequately addressed your comments raised in a previous round of review and you feel that this manuscript is now acceptable for publication, you may indicate that here to bypass the “Comments to the Author” section, enter your conflict of interest statement in the “Confidential to Editor” section, and submit your "Accept" recommendation.

Reviewer #1: All comments have been addressed

Reviewer #2: All comments have been addressed

**********

2. Is the manuscript technically sound, and do the data support the conclusions?

The manuscript must describe a technically sound piece of scientific research with data that supports the conclusions. Experiments must have been conducted rigorously, with appropriate controls, replication, and sample sizes. The conclusions must be drawn appropriately based on the data presented.

Reviewer #1: Yes

Reviewer #2: Yes

**********

3. Has the statistical analysis been performed appropriately and rigorously?

Reviewer #1: Yes

Reviewer #2: Yes

**********

4. Have the authors made all data underlying the findings in their manuscript fully available?

The PLOS Data policy requires authors to make all data underlying the findings described in their manuscript fully available without restriction, with rare exception (please refer to the Data Availability Statement in the manuscript PDF file). The data should be provided as part of the manuscript or its supporting information, or deposited to a public repository. For example, in addition to summary statistics, the data points behind means, medians and variance measures should be available. If there are restrictions on publicly sharing data—e.g. participant privacy or use of data from a third party—those must be specified.

Reviewer #1: Yes

Reviewer #2: Yes

**********

5. Is the manuscript presented in an intelligible fashion and written in standard English?

PLOS ONE does not copyedit accepted manuscripts, so the language in submitted articles must be clear, correct, and unambiguous. Any typographical or grammatical errors should be corrected at revision, so please note any specific errors here.

Reviewer #1: Yes

Reviewer #2: Yes

**********

6. Review Comments to the Author

Please use the space provided to explain your answers to the questions above. You may also include additional comments for the author, including concerns about dual publication, research ethics, or publication ethics. (Please upload your review as an attachment if it exceeds 20,000 characters)

Reviewer #1: We sincerely appreciate all your diligent efforts in thoroughly revising the manuscript in response to the reviewers’ comments. The revised paper has now satisfied the criteria for publication.

Reviewer #2: I thank the authors for their careful revision. All points from my previous report have been satisfactorily addressed: the table numbering (Table 14), the incomplete figure reference, the numerical formatting in the ML-KEM section, and the mapping of revisions in the response letter. The added experiments under realistic IoT workloads (Wi-Fi and BLE) and the combined ML-KEM/ML-DSA evaluation strengthen the practical value of the study.

I am happy to recommend acceptance. Two optional copyediting items, not requiring further review: (1) for consistency with the “303,104 B” convention, the numerical values inside the tables could also use the thousands separator; (2) reference [15] does not appear to be cited in the main text and may be added where relevant or removed.

**********

7. PLOS authors have the option to publish the peer review history of their article (what does this mean?). If published, this will include your full peer review and any attached files.

If you choose “no”, your identity will remain anonymous but your review may still be made public.

**Do you want your identity to be public for this peer review?**  For information about this choice, including consent withdrawal, please see our Privacy Policy.

Reviewer #1: No

Reviewer #2: No

**********

# Editor report: Acceptance letter

**DOI:** 10.1371/journal.pone.0355179.r006

**Authors:** Chun-Wei Yang

PONE-D-26-08044R2

PLOS One

Dear Dr. Karcz,

I'm pleased to inform you that your manuscript has been deemed suitable for publication in PLOS One. Congratulations! Your manuscript is now being handed over to our production team.

At this stage, our production department will prepare your paper for publication. This includes ensuring the following:

* All references, tables, and figures are properly cited

* All relevant supporting information is included in the manuscript submission,

* There are no issues that prevent the paper from being properly typeset

You will receive further instructions from the production team, including instructions on how to review your proof when it is ready. Please keep in mind that we are working through a large volume of accepted articles, so please give us a few days to review your paper and let you know the next and final steps.

Lastly, if your institution or institutions have a press office, please let them know about your upcoming paper now to help maximize its impact. If they'll be preparing press materials, please inform our press team within the next 48 hours. Your manuscript will remain under strict press embargo until 2 pm Eastern Time on the date of publication. For more information, please contact onepress@plos.org.

You will receive an invoice from PLOS for your publication fee after your manuscript has reached the completed accept phase. If you receive an email requesting payment before acceptance or for any other service, this may be a phishing scheme. Learn how to identify phishing emails and protect your accounts at https://explore.plos.org/phishing.

If we can help with anything else, please email us at customercare@plos.org.

Thank you for submitting your work to PLOS ONE and supporting open access.

Kind regards,

PLOS ONE Editorial Office Staff

on behalf of

Professor Chun-Wei Yang

Academic Editor

PLOS One
